# Blockade of SIRPα-CD47 axis by anti-SIRPα antibody enhances anti-tumor activity of DXd antibody-drug conjugates

**DOI:** 10.1371/journal.pone.0304985

**Published:** 2024-06-06

**Authors:** Mayumi Sue, Takuya Tsubaki, Yoko Ishimoto, Shinko Hayashi, Saori Ishida, Takafumi Otsuka, Yoshitaka Isumi, Yumi Kawase, Junko Yamaguchi, Takashi Nakada, Jun Ishiguro, Kensuke Nakamura, Reimi Kawaida, Toshiaki Ohtsuka, Teiji Wada, Toshinori Agatsuma, Norihito Kawasaki

**Affiliations:** 1 Discovery Research Laboratories II, Daiichi Sankyo Co., Ltd., Tokyo, Japan; 2 Modality Research Laboratories III, Daiichi Sankyo Co., Ltd., Tokyo, Japan; 3 Translational Science Department I, Daiichi Sankyo Co., Ltd., Tokyo, Japan; 4 Research Innovation Planning Department, Daiichi Sankyo Co., Ltd., Tokyo, Japan; 5 Discovery Research Laboratories V, Daiichi Sankyo Co., Ltd., Tokyo, Japan; 6 Discovery Research Laboratories I, Daiichi Sankyo Co., Ltd., Tokyo, Japan; 7 Modality Research Laboratories I, Daiichi Sankyo Co., Ltd., Tokyo, Japan; 8 Modality Research Laboratories II, Daiichi Sankyo Co., Ltd., Tokyo, Japan; 9 R&D Division, Daiichi Sankyo Co., Ltd., Tokyo, Japan; City of Hope National Medical Center, UNITED STATES

## Abstract

Signal regulatory protein alpha (SIRPα) is an immune inhibitory receptor on myeloid cells including macrophages and dendritic cells, which binds to CD47, a ubiquitous self-associated molecule. SIRPα-CD47 interaction is exploited by cancer cells to suppress anti-tumor activity of myeloid cells, therefore emerging as a novel immune checkpoint for cancer immunotherapy. In blood cancer, several SIRPα-CD47 blockers have shown encouraging monotherapy activity. However, the anti-tumor activity of SIRPα-CD47 blockers in solid tumors seems limited, suggesting the need for combination therapies to fully exploit the myeloid immune checkpoint in solid tumors. Here we tested whether combination of SIRPα-CD47 blocker with antibody-drug conjugate bearing a topoisomerase I inhibitor DXd (DXd-ADC) would enhance anti-tumor activity in solid tumors. To this end, DS-1103a, a newly developed anti-human SIRPα antibody (Ab), was assessed for the potential combination benefit with datopotamab deruxtecan (Dato-DXd) and trastuzumab deruxtecan (T-DXd), DXd-ADCs targeting human trophoblast cell-surface antigen 2 and human epidermal growth factor receptor 2, respectively. DS-1103a inhibited SIRPα-CD47 interaction and enhanced antibody-dependent cellular phagocytosis of Dato-DXd and T-DXd against human cancer cells. In a whole cancer cell vaccination model, vaccination with DXd-treated cancer cells led to activation of tumor-specific T cells when combined with an anti-mouse SIRPα (anti-mSIRPα) Ab, implying the benefit of combining DXd-ADCs with anti-SIRPα Ab on anti-tumor immunity. Furthermore, in syngeneic mouse models, both Dato-DXd and T-DXd combination with anti-mSIRPα Ab showed stronger anti-tumor activity over the monotherapies. Taken together, this study provides a preclinical rationale of novel therapies for solid tumors combining SIRPα-CD47 blockers with DXd-ADCs.

## Introduction

Myeloid cells represent an abundant cell population in the tumor microenvironment in many types of cancers [1, 2]. While myeloid cells including macrophages and dendritic cells sample antigens and present those to T cells to eradicate the threat during an infection [3], their immune functions toward malignant cells are suppressed in the tumor microenvironment [4]. As such, cancer therapy targeting myeloid cells are of great interest.

One promising pathway is signal regulatory protein alpha (SIRPα)-CD47 interaction between myeloid and tumor cells [5, 6]. SIRPα is a type-I transmembrane protein expressed on various myeloid cells and binds to CD47, a pleiotropic cell surface protein [7–9]. While CD47 interacts with various proteins in the immune system such as thrombospondin-1 and integrins [9], SIRPα-CD47 interaction has been shown to inhibit cellular activation of macrophages such as phagocytosis [7, 10, 11], which is the so called “Don’t-eat-me” signal. In the tumor microenvironment, CD47-SIRPα axis is exploited by cancer cells to suppress the myeloid cell function [12]. Indeed, CD47 expression is upregulated in many types of cancers, not only solid tumors but also blood cancers [13–15], implying the importance of this pathway in the broad cancer biology. These fundamental discoveries led to the development of various SIRPα-CD47 blockers in order to enhance the cancer phagocytosis and antigen presentation to cytotoxic T cells by myeloid cells [5, 6, 16], which subsequently induces anti-tumor immunity. Preclinically, such SIRPα-CD47 blockers enhance antibody-dependent cellular phagocytosis (ADCP) against cancer cells when combined with ADCP-enabling antibodies, such as rituximab, an anti-CD20 antibody (Ab) [17–20].

In blood cancer patients, several SIRPα-CD47 blockers have shown encouraging monotherapy activity, their monotherapy activity in solid tumors seems less promising. In acute myeloid leukemia and B cell lymphoma patients, several CD47 inhibitors resulted in partial response and complete remission (CR) in both monotherapy and combination with the standard of care [16]. Magrolimab, an anti-CD47 Ab, in combination with azacytidine showed 33% CR and 75% objective response rate in untreated patients with higher-risk myelodysplastic syndromes [16]. Unfortunately, despite the high response rate in early phase studies, the development of magrolimab in higher-risk myelodysplastic syndromes and acute myeloid leukemia has been terminated (NCT03248479 and NCT05079230). In solid tumors, SIRPα-CD47 blockers resulted in fewer number of partial response and CR in the early clinical trials [21, 22]. For instance, magrolimab showed a 6.7% objective response rate in previously treated colorectal cancer patients in a combination with cetuximab, an anti-epidermal growth factor receptor Ab [22]. These suggests appropriate combination partners are needed to fully exploit the myeloid immune checkpoint in the solid tumors.

Antibody-drug conjugates (ADCs) bearing a topoisomerase I inhibitor DXd (DXd-ADCs), such as datopotamab deruxtecan (Dato-DXd) and trastuzumab deruxtecan (T-DXd) targeting trophoblast cell-surface antigen 2 (TROP2) and human epidermal growth factor receptor 2 (HER2), respectively, are emerging as a promising therapy for several solid tumors [23, 24]. While both DXd-ADCs have shown monotherapy efficacy in lung and breast cancer patients [25], several combination therapies are being investigated to further enhance the treatment outcomes. In context of immuno-oncology, anti-PD-1 and PD-L1 Abs have been tested as a combination drug for the Dato and T-DXd in lung and breast cancers [26]. Since anti-SIRPα Ab activates the innate arm of anti-tumor immunity, a different mode of action from anti-PD-1 and PD-L1 Abs, we sought to examine whether the combination of SIRPα-CD47 blocker with Dato-DXd or T-DXd would enhance anti-tumor efficacy in solid tumors. Previous studies suggest the cytotoxic payload DXd induces immunogenic cell death (ICD) of the cancer cell [27, 28], potentially activating immune cells in the tumor microenvironment. Furthermore, both Dato-DXd and T-DXd are in a human IgG1 format [23, 24], which in general is capable of inducing ADCP, the myeloid cell function suppressed by the SIRPα-CD47 interaction.

To test this hypothesis, DS-1103a, a novel anti-human SIRPα Ab was established. DS-1103a inhibited SIRPα-CD47 interaction and enhanced ADCP activity of Dato-DXd or T-DXd against human lung or breast cancer cells, highlighting the benefit of the combination. Further, combination of Dato-DXd or T-DXd with an anti-mouse SIRPα Ab, used as a surrogate Ab for DS-1103a as it did not cross-react with mSIRPα, showed enhanced anti-tumor activity compared with the monotherapies. As such, the data form the basis of a novel combination therapy of DS-1103a with DXd-ADCs.

## Materials and methods

### Cells and reagents

Cell lines used in this study are summarized in S1 Table in S1 File. All cell culture was conducted in CO_2_ incubator at 37°C and 5% CO_2_. Cells passaged less than 15 times were used in this study. DS-1103a, a humanized IgG4 Ab against human SIRPα, was generated by humanization of a rat monoclonal Ab against human SIRPα, which was developed by immunization of rats with a human SIRPα protein. Humanization of the rat monoclonal Ab was performed using the complementarity determining region (CDR) grafting method [29]. Briefly, the CDR of the rat monoclonal Ab was grafted into a human IgG framework. Anti-mouse SIRPα monoclonal Ab was generated by immunization of rats with a mSIRPα protein and further chimerized with a mouse IgG1 Fc, resulting in a rat anti-mouse SIRPα mouse IgG1 Fc chimeric Ab. The binding of the anti-mSIRPα Ab to mSIRP family from both C57BL/6 and BALB/c strains and inhibitory effect on mSIRPα-CD47 interaction was validated as shown in S1 Fig in S1 File. A control Ab (human IgG4) against an unrelated antigen was generated and used as an isotype-matched negative control antibody for DS-1103a. For anti-mouse SIRPα Ab, a mouse IgG1 Ab was used as an isotype-matched negative control Ab (Clone MOPC-21, BioLegend, San Diego, CA). Dato-DXd, T-DXd, and the control ADCs were synthesized as previously described [23, 24]. Trastuzumab was purchased (CHUGAI PHARMACEUTICAL, Tokyo, Japan). Datopotamab was prepared as described previously [23]. Ultra-LEAF purified human IgG1 isotype control recombinant antibody (Clone QA16A12, BioLegend) was used as an isotype-matched negative control Ab for trastuzumab and datopotamab.

### Surface plasmon resonance analysis

Surface plasmon resonance analysis was performed with Biacore T200 (Cytiva, Marlborough, MA) by Shin Nippon Biomedical Laboratories (Kagoshima, Japan). Briefly, anti-human IgG (Fc) Ab (Cytiva) was immobilized to the CM5 sensor chip (Cytiva). DS-1103a was captured on the immobilized anti-human IgG (Fc) antibody, and then interaction between immobilized DS-1103a and human SIRPα V1 recombinant protein (R&D systems, Minneapolis, MN) was measured by multi cycle kinetics method. Three assays were performed with the same immobilized anti-human IgG (Fc) antibody, independently.

### Protein conjugation

HRP-conjugated human and mouse CD47-Fc was prepared using HRP Conjugation Kit—Lightning-Link (Abcam, Cambridge, UK) according to the manufacturer’s instruction. Briefly, human and mouse CD47-Fc (R&D Systems) was dissolved in 80 μL of PBS, followed by the addition of 10 μL of Modifier reagent. The human and mouse CD47-Fc/Modifier reagent solution was added to the HRP mix tube, mixed by pipetting, and subsequently incubated for 3 h at room temperature in the dark. Ten microliters of Quencher reagent were added to the mixture and further incubated for 0.5 h at room temperature in the dark. The resultant solution was used as 0.5 mg/mL HRP-conjugated hCD47-Fc or mCD47-Fc solution.

Alexa Fluor 488 (AF488)-labeled DS-1103a was synthesized using AF488 sulfodichlorophenol ester (Thermo Fisher Scientific). Briefly, AF488 sulfodichlorophenol ester (10 mM in dimethyl sulfoxide, 10 eq.) was added to 10 mg/mL DS-1103a in 0.1 M NaHCO_3_ solution. The mixture was incubated for 18 h at 20°C. After the incubation, AF488-labeled DS-1103a was purified by a size-exclusion chromatography with NAP-25 disposable column (CyTiva) equilibrated with 5 mM phosphate buffer pH 7.4, 10% trehalose.

### Cell-based binding assay

To assess the binding of DS-1103a to various human SIRP family proteins, CHO-K1 cells were transiently transfected with pFLAG-myc-CMV-19-DEST encoding SIRPα variant 1 (V1) (NCBI accession #: NP_001035111.1), SIRPα variant 2 (V2) (AAH38510.1), SIRPβ1 (NP_006056.2), or SIRPγ (NP_061026.2) using a FuGENE6 transfection reagent (Promega, Madison, WI) and seeded into a collagen-coated 96 well plate. One day after transfection, after washing with PBS containing 5% FBS (wash buffer, Thermo Fisher Scientific), cells were incubated with DS-1103a or control Ab (human IgG4) for 1 h at 4°C. After washing the wells, cells were incubated with Peroxidase-AffiniPure F(ab’)2 Fragment Goat Anti-Human IgG, Fc gamma Fragment Specific (HRP-conjugated anti-human IgG, Jackson ImmunoResearch, West Grove, PA) for 1 h and at 4°C. After washing, the signal was developed by TMB substrate and STOP solutions (SeraCare Life Sciences, Milford, MA). Absorbance at 450 nm (A450) of each well was measured by SpectraMax M3 (Molecular Devices, San Jose, CA) and SoftMax Pro 6.3 software (Molecular Devices). To assess the inhibitory effect of DS-1103a, the binding of HRP-conjugated hCD47-Fc (final 0.5 μg/mL) to CHO-K1 cells expressing with hSIRPα V1 or V2 in the presence or absence of DS-1103a or control Ab (human IgG4) was analyzed as above.

### ELISA

To assess the binding of anti-mSIRPα mAb, Nunc-Immuno Plate II (Thermo Fisher Scientific) was coated with 0.2 μg/mL solution of various mSIRP family proteins including mSIRPα and mSIRPβ1 of both C57BL/6 (R&D Systems) and BALB/c strains (Sino Biological, Wayne, PA) for overnight at 4°C. The plate was washed with Quantikine ELISA Wash Buffer 1 (R&D Systems) and blocked with Blocker BSA (10×) in PBS (Thermo Fisher Scientific) diluted in PBS for 1 h at room temperature. After washing with the wash buffer, anti-SIRPα mAb or the isotype-matched negative control Ab (mouse IgG1) were added into the wells in triplicate. The plate was incubated for 1 h at room temperature. After washing three times with the wash buffer, the plate was further incubated with Peroxidase AffiniPure Goat Anti-Mouse IgG, Fcγ subclass 1 specific (anti-mIgG1-HRP, Jackson ImmunoResearch) for 30 min at room temperature. After washing the plate with the wash buffer three times, TMB substrate solution (SeraCare Life Sciences) was added to each well, and the assay plate was incubated at room temperature. Subsequently, TMB Stop Solution (SeraCare Life Sciences) was added to each well to stop the reaction. A450 of each well was measured by Ensight plate reader (PerkinElmer, Waltham, MA).

To assess the inhibitory effect of anti-mSIRPα mAb on mCD47-mSIRPα interaction, the ELISA assay was performed as above. Briefly, mSIRPα of both C57BL/6 and BALB/c strains were coated on the ELISA plate. Anti-mSIRPα or isotype-control Ab (human IgG4) was added, followed by the addition of HRP-conjugated mCD47-Fc (final 50 ng/mL).

### Preparation of human monocyte-derived macrophages

Human peripheral blood was obtained in accordance with the guidelines of the Daiichi Sankyo Research Ethics Committee (Approval No. 000033). The study started on 10 Oct 2019 and completed on 13 June 2023. Written informed consent was obtained from all anonymized donors. The obtained blood samples were used according to the approved protocol and no samples were stored for any other use. Peripheral blood mononuclear cells (PBMCs) were isolated using Ficoll-Paque PLUS (CyTiva) and SepMate-50 tubes (STEMCELL Technologies, Vancouver, Canada). Monocytes were further purified from PBMCs using an EasySep Human Monocyte Enrichment Kit without CD16 Depletion (STEMCELL Technologies) according to the manufacturer’s instruction. Purified monocytes were resuspended in RPMI 1640 (Thermo Fisher Scientific) supplemented with 10% heat-inactivated fetal bovine serum (FBS, GE Healthcare, Chicago, IL) (R10) containing 20 ng/mL recombinant human M-CSF (PeproTech, Cranbury, NJ) and cultured for 7 days in a 5% CO_2_ incubator at 37°C. On day 7, the medium was replaced with fresh R10 containing 20 ng/mL recombinant human M-CSF and further cultured for 4 days. On day 11, the medium was replaced again with fresh R10 containing 20 ng/mL recombinant human M-CSF and recombinant human IL-10 (PeproTech). The cells were cultured for 2 days for M2 macrophage polarization. On day 13, the adherent cells were harvested and used as human macrophages for the experiments in this study.

### Evaluation of DS-1103a binding to human macrophages

To assess DS-1103a binding to human macrophages, 1 x 10^5^ cells of human macrophages obtained from blood monocytes were incubated with LIVE/DEAD-NIR (Thermo Fisher Scientific) for 30 min at 4°C in the dark. After washing the cells with Stain buffer (FBS) (BD Biosciences, Franklin Lakes, NJ), the cells were incubated with BD Fc Block human (BD Biosciences) for 10 min at room temperature. Cells were further incubated with AF488-labeled DS-1103a for 1 h at 4°C. The stained cells were washed and fixed with Stabilizing Fixative 3× Concentrate (BD Biosciences) for 30 min at 4°C. Fixed cells were analyzed with BD LSRFortessa X-20 (BD Biosciences) and the obtained data were analyzed with FlowJo software (FlowJo, Ashland, OR). To quantify DS-1103a binding to macrophages, anti-IgG, Human, Goat-Poly, Microsphere, Quantum Simply Cellular (Bangs Laboratories, Technology Drive Fishers, IN) was used.

### Evaluation of ADCP

Human macrophages and human cancer cell lines (LK-2 and SK-BR-3) were fluorescently labeled with the CellTrace Far Red, Violet, or carboxyfluorescein succinimidyl ester (CFSE) solution (Thermo Fisher Scientific) according to the manufacturer’s instruction. The fluorescently labeled cancer cells (5 x 10^4^ cells/well) were seeded in a 96-well U-bottom plate (PrimeSurface 96U, Sumitomo Bakelite, Tokyo, Japan), followed by the addition of the indicated reagents. The fluorescently labeled macrophages (5 x 10^4^ cells/well) were added to the wells, mixed gently, and incubated for 4 h at 37°C. The cells were washed with PBS or Hanks’ Balanced Salt Solution containing 2 or 0.1% BSA and fixed with Stabilizing Fixative 3× Concentrate. Fixed cells were analyzed using BD LSRFortessa X-20 or Attune NxT acoustic focusing cytometer (Thermo Fisher Scientific). The obtained data were analyzed with FlowJo software. The gating strategy for ADCP analysis was shown in S2 Fig in S1 File.

### ICD induction

HCC827 (4 x 10^4^ cells/well), SK-BR-3 (2 x 10^4^ cells/well), CT26.WT (1 x 10^6^ cells/well), or hTROP2-MC38 cells (2 x 10^4^ cells/well) were seeded in a 96-well plate or 6-well plate for CT26.WT cells. On the following day, the supernatant was removed and the indicated reagent or Dimethyl sulfoxide (DMSO) alone was added. The cells were incubated at 37°C for the indicated time period and extracellular HMGB1, extracellular ATP (eATP), and cell viability was monitored. For HMGB1 detection, Lumit HMGB1 Immunoassay (Promega) or HMGB1 ELISA kit II (SHINO-TEST, Tokyo, Japan) were used. For eATP detection, RealTime-Glo Extracellular ATP Assay (Promega) or BacTiter-Glo Reagent (Promega) were used, and the signals were expressed as relative light unit (RLU). For cell viability measurement, CellTiter-Glo Luminescent Cell Viability Assay (Promega) were used. The luminescent signals were detected using Ensight (PerkinElmer) or Envision (PerkinElmer) plate readers.

### Animal studies

All animal experiments were conducted in accordance with the guidelines of the Institutional Animal Care and Use Committee at Daiichi Sankyo (Approval No. 20000191 and 21000548). All animals were euthanized either by CO_2_ or by exsanguination under 2% isoflurane (Mylan Seiyaku, Tokyo, Japan) anesthesia. The treatment groups of animals were not masked to the researchers. When power analysis was employed to determine the sample size of animal experiments, the significant level and power was set to be 0.05 and 0.80, respectively.

To assess the impact of ICD on the immune activation *in vivo*, cancer vaccination study was performed. Briefly, on the day before vaccination, CT26.WT cells were treated with 4 μM of DXd. On day 0, 5 x 10^5^ of the CT26.WT cells were injected subcutaneously to six-weeks old female BALB/c mice in the right flank. As a negative control immunogen with no ICD, CT26.WT cells treated with repeated freezing and thawing (necrotic cell death, NCD) were used. Mice were randomized to the vehicle control and the treatment groups and received the following treatments (n = 6 for each group). Anti-mSIRPα Ab (10 mg/kg), anti-mouse CD47 (mCD47) Ab (10 mg/kg, clone MIAP410, Bio X Cell, Lebanon, NH), or PBS was injected intraperitoneally on days 1 and 5. On day 7, the animals were inoculated with 3 x 10^6^ of intact CT26.WT cells in the left flank. On day 21, the tumor volume defined as 1/2 × (length) × (width)^2^ was measured. To further analyze the impact of ICD on immune activation, on day 33, animals were euthanized, and the spleens were harvested and analyzed for T cell activation by enzyme-linked immunosorbent spot (ELISPOT) assay using mouse IFN-gamma single-color ELISPOT 96-well white precoated (Cellular Technology, Shaker Heights, OH) according to the manufacturer’s instruction. Briefly, 1 x 10^5^ of splenocytes were incubated with the same number of CT26.WT cells as a target. The number of spots were counted using ELISPOT analyzer S6 Entry M2 (Cellular Technology).

To assess the combination anti-tumor effect of anti-mSIRPα Ab with Dato-DXd *in vivo*, five-weeks old female C57BL/6 mice were inoculated subcutaneously with 1 x 10^6^ of hTROP2-MC38 cells in the right flank on day 0. On day 7 when the tumor volume reached approximately 190 mm^3^, mice were randomized to the vehicle control group and the treatment groups and received the following treatments (n = 20 each group). The mice excluded from the study due to the variation in tumor volume at the randomization was euthanized by CO_2_. Anti-mSIRPα Ab (10 mg/kg) or PBS was injected intraperitoneally. Dato-DXd (1 mg/kg) or 10 mM-Acetate Buffer, 5% sorbitol, pH 5.5 was injected intravenously. The tumor volume defined as above was measured twice a week. To analyze the tumor-infiltrating lymphocytes, on day 15 all animals were euthanized, and the tumor was harvested and processed as described previously [28]. Briefly, tumors were cut into small pieces and digested with tumor dissociation Kit (Miltenyi Biotec, Bergisch Gladbach, Germany) using gentleMACS Octo Dissociator with Heaters (Miltenyi Biotec). After the lysis of red blood cells with BD Pharm Lyse Lysing buffer (BD Biosciences), One million of the obtained cells from each animal were stained for both cell surface and intracellular antigens using the antibodies listed in S2 Table in S1 File. The stained cells were analyzed by BD LSRFortessa X-20 and the obtained data were analyzed with FlowJo software. The gating strategy of TIL analysis is shown in S3 Fig in S1 File.

The combination study of T-DXd with anti-mSIRPα Ab was conducted at LSIM Safety Institute Corporation (Kumamoto, Japan) in accordance with the guidelines of the Institutional Animal Care and Use Committee at LSIM Safety Institute Corporation (Approval No. 2021–0417). To evaluate the combination anti-tumor effect of anti-SIRPα Ab with T-DXd *in vivo*, six-weeks old female BALB/c mice were anesthetized by inhalation of 2% isoflurane and inoculated subcutaneously with 3 x 10^6^ of hHER2-CT26.WT cells in the right flank on day 0. On day 6 when the tumor volume reached approximately 110 mm^3^, mice were randomized as described above and received the following treatments (n = 15 for each group). Anti-mSIRPα Ab (10 mg/kg) or saline was injected intraperitoneally on days 6, 9, 13, and 16. T-DXd (5 mg/kg) was injected intravenously on days 6 and 13 in combination with anti-mSIRPα Ab or saline (monotherapy). Trastuzumab (15 mg/kg) or vehicle alone was injected intravenously on days 6, 9, 13, and 16 in combination with anti-mSIRPα Ab or saline (monotherapy). The tumor volume was measured as above and animals were euthanized when reached a humane endpoint.

To monitor SIRPα receptor occupancy (RO) by anti-mSIRPα Ab when administered in mice, anti-mSIRPα Ab (10 mg/kg) was intraperitoneally administered into BALB/c mice on day 0. Just before administration, and on 1, 3, 7, and 10 days after administration, peripheral blood were collected from the heart of each of the mice using a heparinized syringe under 2 to 3% isoflurane inhalation anesthesia. After blood sampling, the mice were euthanized by exsanguination under the anesthetic conditions indicated above. The obtained blood samples were centrifuged at 1000 ×g for 10 min at 25°C. The blood cell pellet from each sample was used to evaluate receptor occupancy by flow cytometry. Briefly, after red blood cell lysis, cells were stained with three different combinations of Abs as below; #1 Brilliant Violet 421 anti-mouse Ly-6G/Ly-6C (Gr-1) Ab (BioLegend, 1:100 dilution, clone RB6-8C5) and PerCP/Cyanine5.5 anti-mouse CD11c Ab (BioLegend, 1:40 dilution, clone N418), #2 Gr-1 Ab, CD11c Ab, and PE goat anti-mouse IgG (minimal x-reactivity) Ab (BioLegend, 1:100 dilution), #3 Gr-1 Ab, CD11c Ab, and PE goat anti-mouse IgG, and anti-mSIRPα Ab. For the staining #3, cells were first stained with anti-mSIRPα Ab, followed by the other antibodies. For the staining #1 and 2, staining was performed at one step. The stained cells were washed with PBS containing 5% FBS (GE Healthcare) and 1 mM EDTA (Nacalai Tesque, Tokyo, Japan), fixed with Stabilizing Fixative 3× Concentrate at 4°C, and analyzed by BD FACSCanto II (BD Biosciences). Mean fluorescence intensity (MFI) of each staining was calculated in FlowJo software and SIRPα RO was calculated as described formula below.


$$RO\;[\%]=[\frac{\left(PE\;MFI\;of\;\#2‐PE\;MFI\;of\;\#1\right)}{\left(PE\;MFI\;of\;\#3‐PE\;MFI\;of\;\#1\right)}]\times 100$$

### Statistical analysis

All statistical analyses were performed in SAS System version 9.2 (SAS Institute Inc, Cary, NC). Paired sample t-test (Figs 2B and 6A), Dunnett’s multiple comparison (Figs 4A and 4B, 5, and 6C left), Tukey’s multiple comparison (Fig 4C and 4D) and log-rank test (Fig 6C right) were employed. The estimated sigmoid curves were drawn using GraphPad PRISM (GraphPad Software, LLC., version 9.1.0).

## Results

### DS-1103a, a novel anti-human SIRPα antibody, binds to human SIRPα V1 and V2 and inhibits SIRPα-CD47 interaction

**Fig 1:**
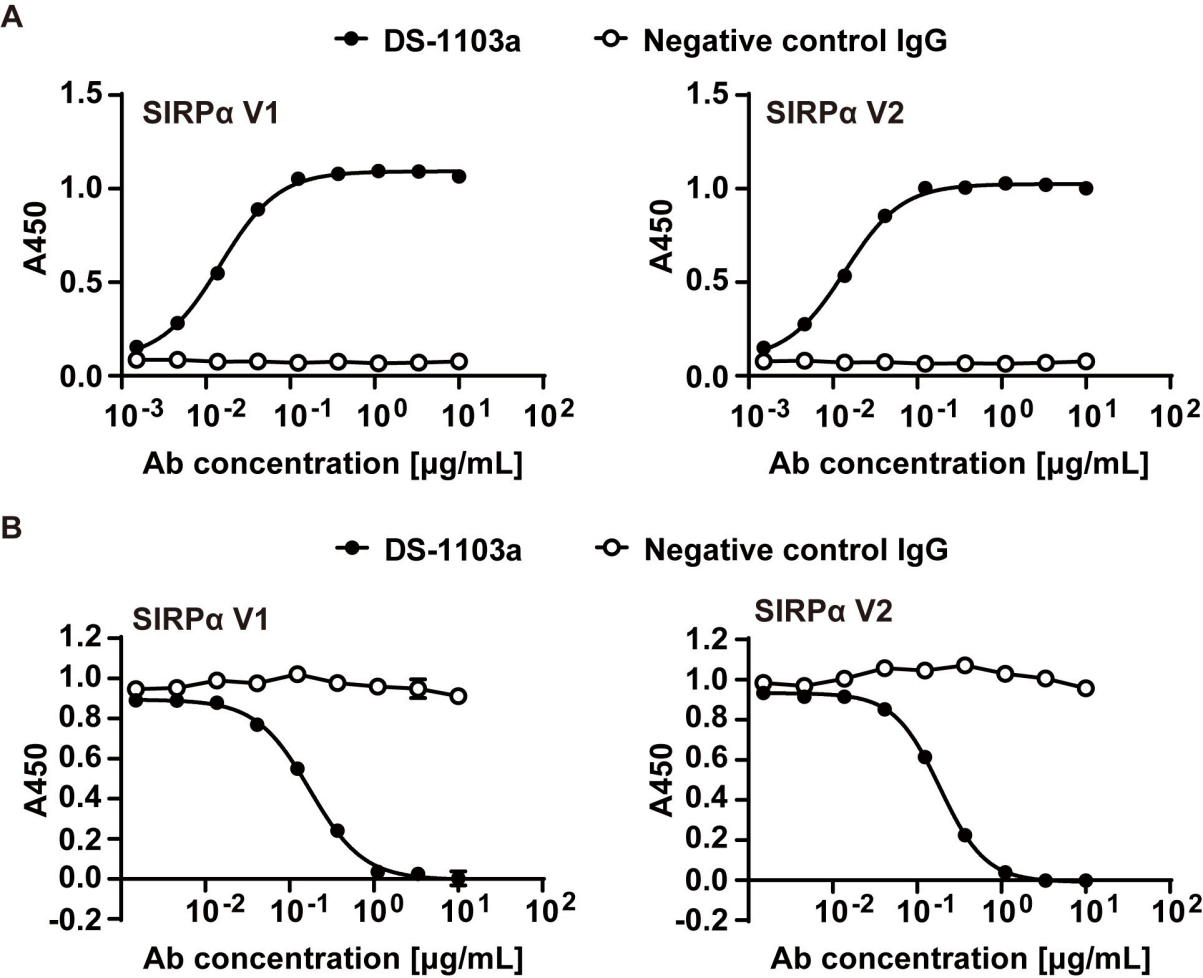
DS-1103a binds to human SIRPαV1 and V2 and inhibits SIRPα-CD47 interaction. (**A**) The binding of DS-1103a or negative control human IgG4 to human SIRPαV1 and V2 proteins expressed on CHO-K1 is shown. The binding was measured in a cell-based assay by monitoring A450. Each data point represents the mean ± standard error of A450 from three technical replicates. (**B**) CD47-Fc binding to human SIRPαV1 and V2 proteins expressed on CHO-K1 in the presence of DS-1103a or negative control human IgG4 is shown. Competition assay was performed using the binding assay described in (**A**). Representative data from two independent experiments are shown.

DS-1103a, an anti-human SIRPα Ab was developed. DS-1103a bound to human SIRPα variants V1 and V2, the major variants [19, 30], in a concentration-dependent manner, whereas isotype-matched negative control Ab (human IgG4) did not (Fig 1A). DS-1103a also bound to other SIRP family molecules including human SIRPβ1 and SIRPγ (S4 Fig in S1 File). Further, the inhibitory activity of DS-1103a on SIRPα-CD47 interaction was assessed using human SIRPα V1 or V2-expressing CHO-K1 and HRP-conjugated CD47-Fc. The interaction between human SIRPα and CD47 was inhibited by DS-1103a in a concentration-dependent fashion, which was not seen in the negative control Ab (human IgG4) (Fig 1B). From surface plasmon resonance analysis, DS-1103a was shown to bind to human SIRPα V1 at a k_on_ of 8.08 ± 0.55 x 10^5^ [M^-1^s^-1^] and a k_off_ of 4.98 ± 0.14 x 10 ^-4^ [s^-1^]. These results indicate DS-1103a binds to human SIRPα major variants V1 and V2 and inhibits SIRPα-CD47 interaction.

### DS-1103a binds to human monocyte-derived macrophages and enhances ADCP of Dato-DXd

**Fig 2:**
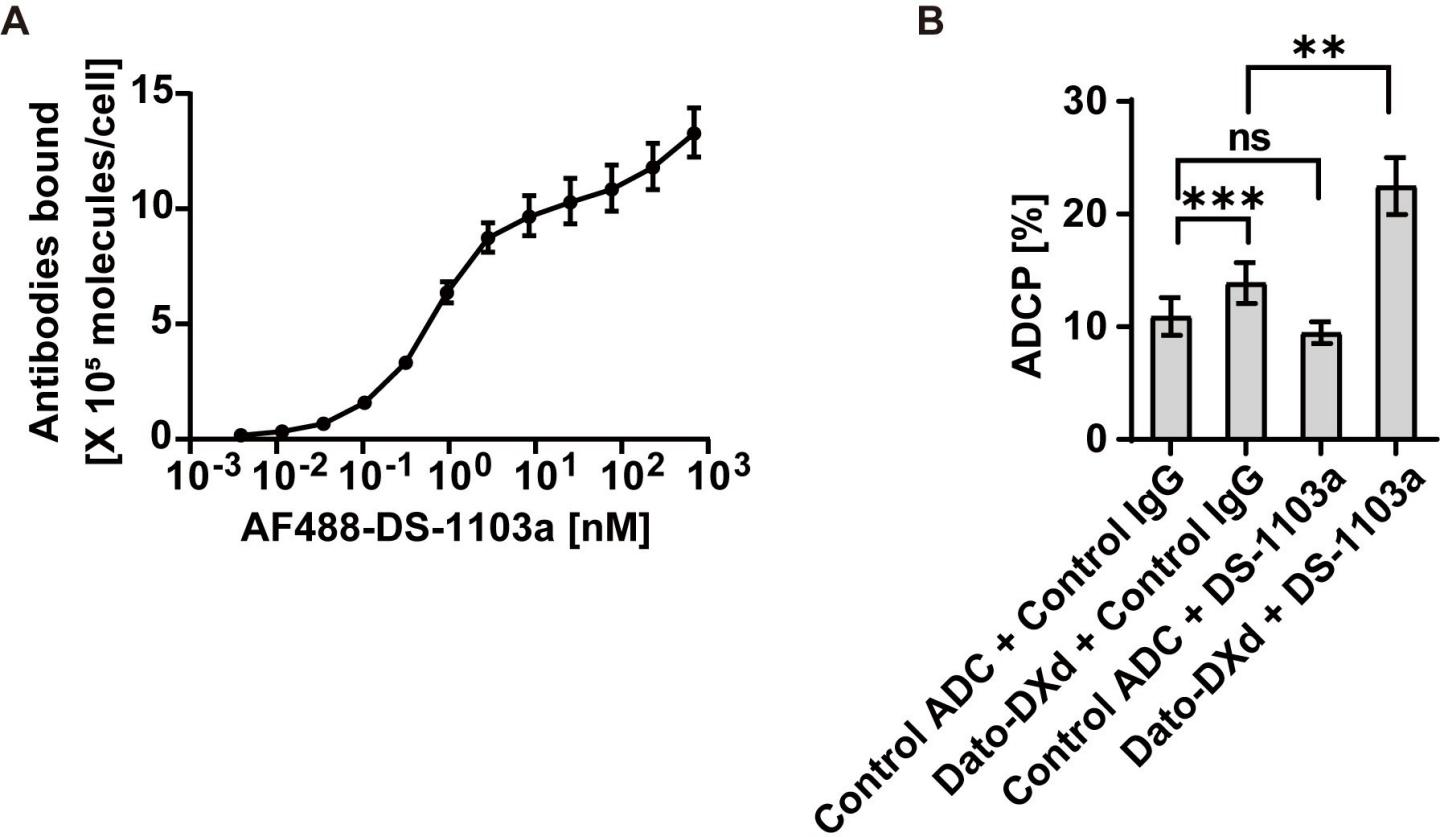
DS-1103a binds to human macrophages and enhances the ADCP of Dato-DXd against human lung cancer cells. (**A**) The binding of AF488-labeled DS-1103a to human monocyte-derived macrophages was analyzed by flow cytometry. The number of antibodies bound per cell was estimated using the calibration beads and is shown as geomean and geoSD (n = 3). (**B**) Far Red-labeled macrophages were co-cultured with Violet-labeled LK-2 cells and ADCP activity [%] was measured by flow cytometry. The data represent the mean ± standard error (n = 4). P-values were determined by the paired-sample t-test. ***, **, and ns indicate P < 0.001, P < 0.01, and not significant, respectively. Representative data from two independent experiments are shown.

To test whether DS-1103a enhances ADCP of Dato-DXd against human lung cancer cells, human monocytes from healthy individuals were cultured with human M-CSF and IL-10 to induce M2-like macrophages *in vitro*. AF488-labeled DS-1103a successfully bound to the macrophages, confirming SIRPα expression on the macrophages used (Fig 2A). When the macrophages were co-cultured with LK-2 cells, a human lung cancer cell line expressing TROP2 [23], Dato-DXd induced ADCP against LK-2 cells (Fig 2B), compared with the control ADC. On the other hand, DS-1103a alone did not induce the ADCP, which was consistent with other anti-SIRPα Abs previously reported [5, 20, 31]. When both Dato-DXd and DS-1103a were added to the co-culture, the ADCP was significantly enhanced, compared with Dato-DXd single treatment (Fig 2B). These data suggest that blocking of SIRPα-CD47 interaction by DS-1103a enhances ADCP activity of Dato-DXd in SIRPα positive macrophages, implying the potential of Dato-DXd as a combination drug for DS-1103a.

### Dato-DXd treatment induces a release of HMGB1 from tumor cells

**Fig 3:**
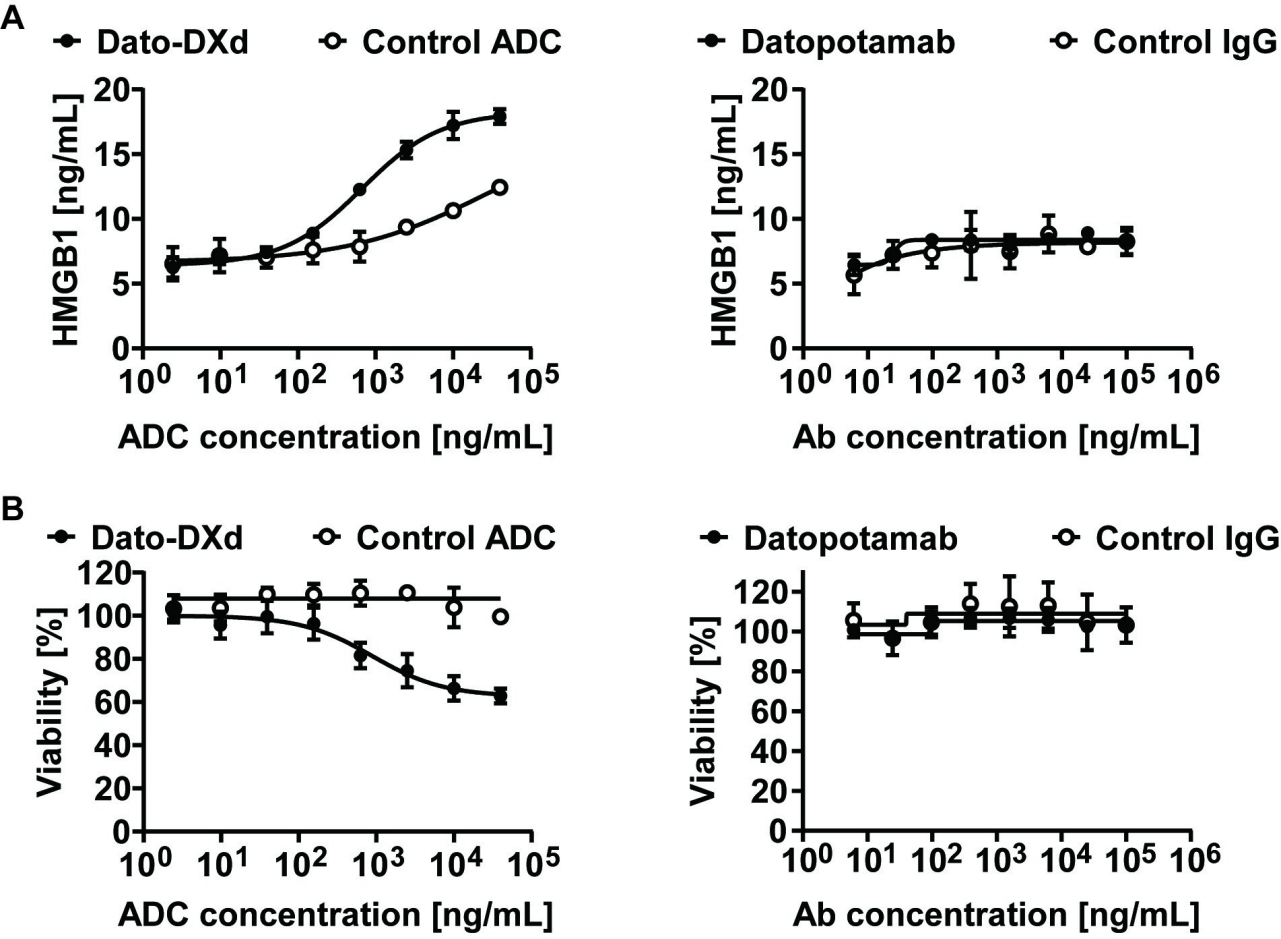
Dato-DXd induces ICD of human lung cancer cells. HCC827 cells were treated with various concentration of Dato-DXd, datopotamab, or the negative control for 7 days. HMGB1 secretion (**A**) and cell viability (**B**) of HCC827 cells were measured by a luminescence-based assay. The data represent the mean ± standard deviation (three technical replicates). Representative data from two independent experiments are shown.

To assess whether Dato-DXd induces ICD *in vitro*, HCC827 cells, a TROP2-positive lung cell line [23], were cultured in the presence of Dato-DXd, datopotamab, control ADC, or control IgG (human IgG1), and HMGB1, a hallmark of ICD [32, 33], secreted from HCC827 was measured. Dato-DXd induced HMGB1 in a concentration-dependent manner (Fig 3A), accompanied with the decreased viability of the cells (Fig 3B), while the control ADC did not. Of note, datopotamab alone failed to show HMGB1 release and cytotoxicity to the cells (Fig 3A and 3B). These results clearly demonstrate Dato-DXd is indeed capable of inducing ICD via the DXd payload, but not the antibody portion.

### Combination treatment of DXd with anti-mSIRPα antibody enhances anti-tumor T cell response

**Fig 4:**
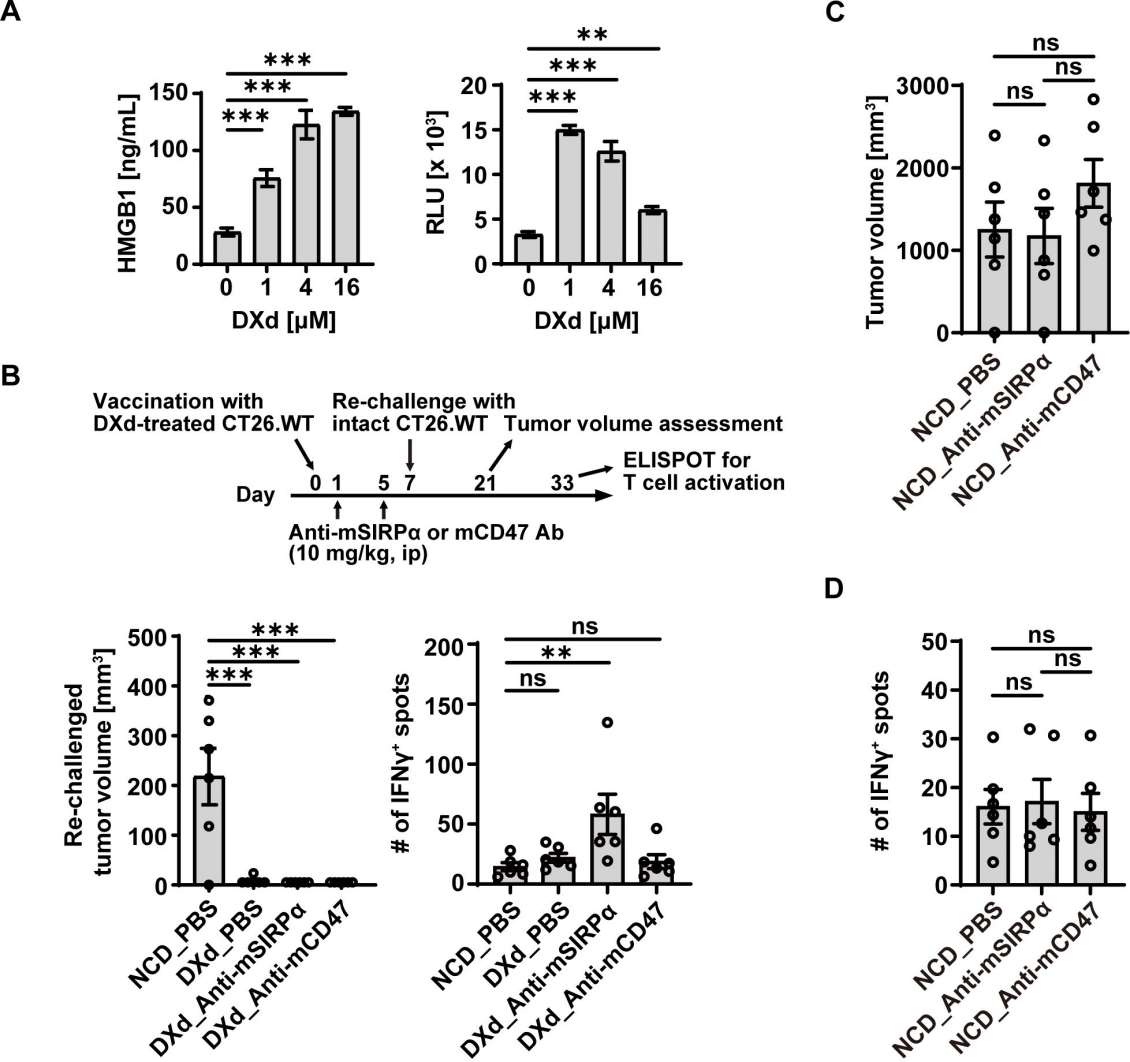
ICD induction by DXd payload enhances anti-tumor T cell response in the presence of anti-mSIRPα Ab. (**A**) CT26.WT cells were cultured with the various concentrations of DXd for 24h, and HMGB1 (left) and eATP (right) secretion was measured by an ELISA and a luminescence measurement. The data represent the mean ± SD (three technical replicates). (**B**) The volume of re-challenged tumor and the T cell reactivity to the tumor in the spleen were monitored in a cancer vaccination study described in materials and methods (n = 6 for each group). (**C** and **D**) In a separate control experiment, naïve mice were immunized with CT26.WT cells treated with freezing and thawing (NCD) on day 0, followed by administration of the indicated reagents and re-challenge with intact CT26.WT cells (n = 6). The volume of re-challenged tumor and the T cell reactivity in the spleen are shown. P values were determined by the parametric Dunnett’s multiple comparison test (**A** and **B**) and parametric Tukey’s multiple comparison test (**C** and **D**). Representative data from two independent experiments are shown.

To evaluate the impact of the ICD induction and SIRPα-CD47 blockade on the subsequent immune response against tumor cells, an anti-mSIRPα Ab was used as DS-1103a did not cross-react to mouse SIRPα and a mouse vaccination model was employed [34]. First, CT26.WT mouse colon carcinoma cells were treated with the DXd payload alone for 24 h which resulted in secretion of HMGB1 and eATP (Fig 4A). The naïve mice were then inoculated with the DXd-treated CT26.WT cells or cells with repeated freezing and thawing (NCD) as a vaccination on day 0 (Fig 4B). To test the potential benefit of combining SIRPα-CD47 blocker with the vaccination, the vaccinated mice were administered with anti-mouse SIRPα or anti-mouse CD47 Ab. A dose at 10 mg/kg for anti-mouse SIRPα Ab was employed throughout this study, as the single administration of the anti-mouse SIRPα Ab at 10 mg/kg achieved around 90% of SIRPα RO on blood myeloid cells in mice (S5 Fig in S1 File). The vaccinated mice were inoculated with intact CT26.WT cells on day 7 (tumor cell re-challenge). To assess the vaccination efficacy, the re-challenged tumor volume was measured on day 21. Furthermore, *ex vivo* reactivity of splenocytes to cancer cells was analyzed to test anti-tumor T cell responses on day 33 (Fig 4B). While the re-challenged tumor grew in the animals with NCD-CT26.WT vaccination, the groups that received DXd-treated CT26.WT vaccination showed significantly reduced tumor volume, some of which showed complete rejection (Fig 4B). Importantly when the isolated splenocytes from the re-challenged animals were tested for their reactivity to cancer cells *ex vivo*, DXd-treated CT26.WT vaccination with anti-mSIRPα Ab administration significantly increased the number of IFNγ-secreting cells, compared with NCD-CT26.WT vaccination group (Fig 4B). Interestingly, the combination of DXd-treated CT26.WT vaccination with anti-mCD47 Ab administration failed to induce T cell activation (Fig 4B). In a separate control experiment, when mice were vaccinated with NCD-CT26.WT cells, both anti-mSIRPα and anti-mCD47 Abs failed to suppress re-challenged tumor growth and induce the T cell activation (Fig 4C and 4D). The lack of T cell activation by anti-mSIRPα Ab without ICD was consistent with the lack of ADCP activity in DS-1103a single treatment in Fig 2B, suggesting DS-1103a requires combination drugs to fully activate the immune system. Taken together, these results highlight the potential benefit of combining DXd with anti-SIRPα Ab to enhance tumor-specific T cell response.

### Combination of Dato-DXd with anti-mSIRPα Ab showed enhanced anti-tumor activity accompanied by T cell activation in the tumor

**Fig 5:**
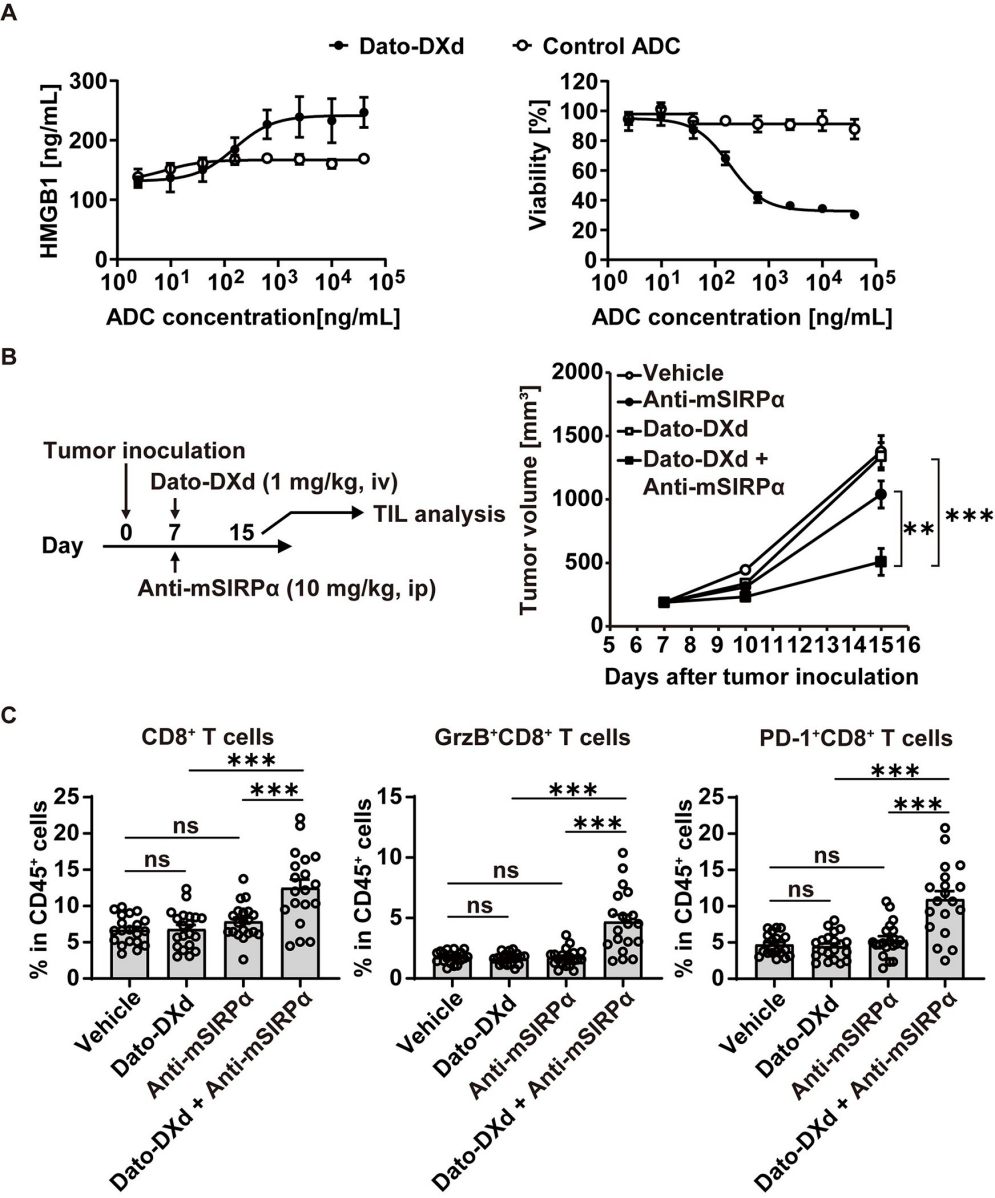
Combination of Dato-DXd with anti-mSIRPα Ab enhances anti-tumor activity, accompanied by T cell activation in the tumor. (**A**) hTROP2-MC38 cells were cultured with the various concentrations of Dato-DXd or control ADC for 4 days. HMGB1 secretion and cell viability of hTROP2-MC38 cells were measured as in Fig 3. The data represent the mean ± SD (three technical replicates). (**B**) hTROP2-MC38 cells were subcutaneously inoculated into the right flank of C57BL/6 mice on day 0. The indicated reagents were administered as a monotherapy or in combination to hTROP2-MC38-bearing mice on day 7. Tumor volume was measured on days 7, 10, and 15, and is shown as mean ± SEM (n = 20 for each group). P values for the tumor volumes on day 15 were determined by parametric Dunnett’s multiple comparison test. (**C**) On day 15, tumors were excised and ground to cell suspensions for tumor-infiltrating lymphocytes analysis by flow cytometry. The frequencies of CD8^+^ T cells, GrzB^+^ CD8^+^ T cells, PD-1^+^ CD8^+^ T cells in CD45^+^ cells from each tumor sample are shown with the mean ± SEM of each group (combination group, n = 19; other groups, n = 20). P values for the frequencies were determined by parametric Dunnett’s multiple comparison test. Representative data from two independent experiments are shown.

The results from the cancer vaccination model led us to test the anti-tumor effect as well as the immune activation of the combination of DXd-ADCs with anti-mSIRPα Ab. To this end, the combination of Dato-DXd with anti-mSIRPα Ab in an immune-competent syngeneic mouse model bearing MC38 cells expressing human TROP2 (hTROP2-MC38) was investigated. hTROP2-MC38 secreted HMGB1 upon Dato-DXd but not control ADC treatment *in vitro* (Fig 5A), verifying this model recapitulates the ICD induction observed in HCC827 cells (Fig 3A). To assess the anti-tumor effect, C57BL/6 mice were inoculated with hTROP2-MC38 on day 0 and received administration of Dato-DXd and anti-mSIRPα Ab on day 7. Tumor volume was monitored until day 15, on which tumors were harvested and analyzed by flow cytometry to assess tumor infiltrating lymphocytes (TIL) activation (Fig 5B and S3 Fig in S1 File). Combination of Dato-DXd with anti-mSIRPα Ab demonstrated stronger anti-tumor effects, compared with each Dato-DXd or anti-mSIRPα antibody alone (Fig 5B). Animals in both combination group of Dato-DXd with anti-mSIRPα Ab and anti-mSIRPα Ab single treatment group showed no abnormality in the body weight changes during the study (data not shown). In the TIL analysis, combination of Dato-DXd with anti-SIRPα Ab demonstrated significant increase of CD8^+^ T cells and granzyme B (GrzB)^+^ CD8^+^ T cells in the tumor (Fig 5C and S6 Fig in S1 File). Furthermore, the expression of PD-1, a T cell activation marker [35], was also increased in the CD8^+^ T cells treated with the combination of Dato-DXd with anti-mSIRPα antibody (Fig 5C and S6 Fig in S1 File). These results indicate that the combination of Dato-DXd with anti-mSIRPα Ab enhances anti-tumor activity accompanied by the activation of CD8^+^ T cells in the tumor.

### Anti-SIRPα antibody enhances the ADCP and anti-tumor activity of T-DXd

**Fig 6:**
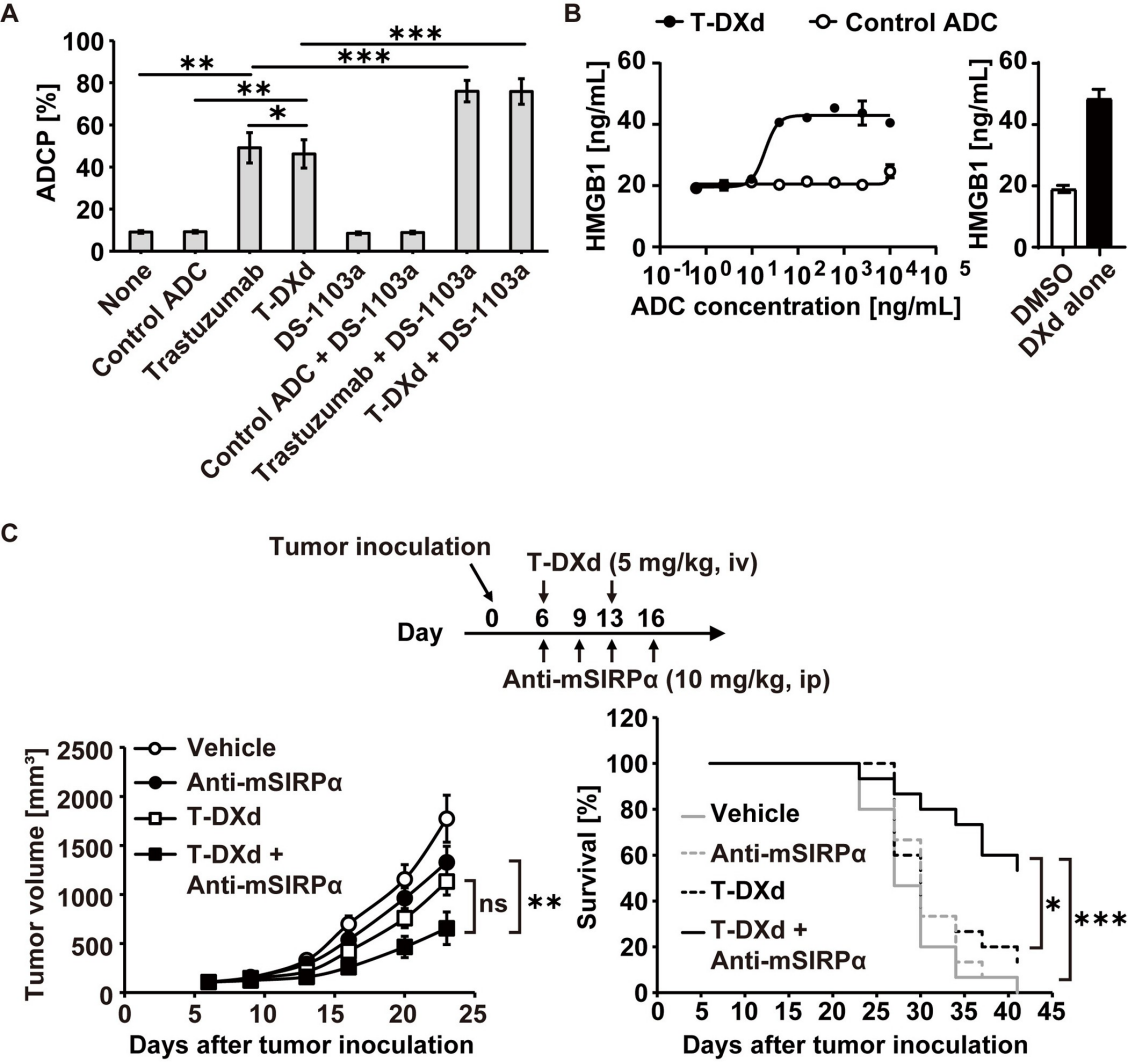
Combination of T-DXd with anti-SIRPα Ab enhances the ADCP and anti-tumor activity of T-DXd. (**A**) ADCP of T-DXd and trastuzumab against SK-BR-3 cells was measured in the presence or absence of DS-1103a as shown in Fig 2. Statistical significance was determined by a two-tailed paired t-test (*: P < 0.05, **: P < 0.01, and ***: P < 0.001). The data represent the mean ± SEM from six healthy donors. (**B**) SK-BR-3 cells were cultured with the various concentrations of T-DXd or control ADC for 4 days. HMGB1 secretion from SK-BR-3 cells was measured as in Fig 3. The data represent the mean ± SD (three technical replicates). (C) hHER2-CT26.WT cells (3 × 10^6^ cells/head) were subcutaneously inoculated into the right flank of BALB/c mice on day 0. The indicated reagents were administered as a monotherapy or in the indicated combinations, starting on day 6. Tumor volumes are shown as mean ± SEM (n = 15). Statistical significance for the tumor volumes on day 23 and survival of the animals were determined by Dunnett’s test and Log-rank test, respectively (*: P < 0.05, **: P < 0.01, and ***: P < 0.001). Representative data from two independent experiments are shown.

Since there are multiple DXd-ADCs being tested in clinical trials, including T-DXd, a HER2-targeting ADC, the combination of anti-SIRPα Ab was assessed to enhance the anti-tumor activity of T-DXd. As shown in Fig 6A, T-DXd and trastuzumab exhibited ADCP against human HER2-positive breast cancer SK-BR-3 cells to a similar extent, which were enhanced by the combination with DS-1103a. T-DXd induced HMGB1 and eATP release from SK-BR-3 cells with the decreased cell viability (Fig 6B and S7 Fig in S1 File) at the concentrations below the reported trough serum concentration of T-DXd in breast cancer patients [36]. On the other hand, while trastuzumab treatment of SK-BR-3 cells resulted in modest reduction in cell viability, it did not lead to the release of HMGB1 and eATP (S7 Fig in S1 File), implying that T-DXd rather than trastuzumab is a better combination drug for anti-SIRPα Ab. To further verify this, the anti-tumor activity of anti-mSIRPα Ab combination with T-DXd or trastuzumab was tested in a mouse model bearing human HER2-expressing CT26.WT (hHER2-CT26.WT). BALB/C mice were inoculated with hHER2-CT26.WT cells and received the indicated treatment from day 6. Combination of T-DXd with anti-mSIRPα antibody significantly extended survival of the animals compared with T-DXd or anti-mSIRPα antibody alone (Fig 6C). Animals in both combination group of T-DXd with anti-mSIRPα Ab and anti-mSIRPα Ab single treatment group showed no abnormality in the body weight changes during the study (data not shown). On the other hand, the combination of trastuzumab with anti-mSIRPα antibody showed no benefit in anti-tumor activity and survival (S8 Fig in S1 File). Overall, these results suggest that the dual function of T-DXd, i.e. ADCP by the antibody portion and ICD by the payload, makes it a promising combination partner for anti-SIRPα Ab to maximize anti-tumor efficacy by activating anti-tumor immunity.

## Discussion

In this report, we described DS-1103a, a novel anti-human SIRPα antibody, and provided a preclinical rationale for the combination therapy of DS-1103a with two DXd-ADCs for solid tumors. DS-1103a has an IgG4 Fc format, an effectorless isotype, to minimize unwanted immune attack to SIRPα-expressing myeloid cells. For this regard, van Helden MJ *et al* showed that anti-human SIRPα Ab in IgG1 isotype induced ADCP against SIRPα-expressing myeloid cells, while the Ab in IgG1 containing L234A/L235A mutations did not [37]. Further, Sim J *et al* showed that anti-human SIRPα Ab clone 21 enhanced cetuximab-induced ADCP both with and without Fc fragment of the anti-SIRPα Ab [38], suggesting the blocking of SIRPα-CD47 interaction is sufficient to enhance ADCP. It is well-known that SIRPα-CD47 interaction occurs both in *trans* and *cis* orientation, the latter occurs in monocytes and other myeloid cells where both SIRPα and CD47 are expressed on the same cell surface [9, 19, 39]. Despite the potential cis-interaction, DS-1103a successfully bound to human macrophages and enhanced the ADCP of Dato-DXd and T-DXd (Fig 2B and 6A). Several biologics targeting SIRPα-CD47 pathway have been developed and shown promising anti-tumor activity in hematological malignancies. For instance, in blood cancer, agents targeting CD47 such as magrolimab, evorpacept, and ontorpacept, and those targeting SIRPα such as CC-95251 have shown anti-cancer activity in monotherapy or combination with the standard of care [6, 16, 40, 41]. In solid tumors, however, the anti-tumor activity of SIRPα-CD47 blockers remains to be improved [6, 21, 42–44]. The combination therapy proposed in this study might enhance anti-tumor activity by two mechanisms as follows. First, DS-1103a was shown to enhance the ADCP activity of both Dato-DXd and T-DXd. Second, ICD induction by the DXd payload has shown synergy with anti-mSIRPα Ab to induce robust T cell activation *in vivo*. As such, this study paves the way to test a new combination therapy for solid tumors, fully harnessing the potential of the SIRPα-CD47 pathway.

In the combination study of Dato-DXd with anti-mSIRPα Ab *in vivo*, the enhanced CD8^+^ T cell activation was observed in the tumor. Of interest, Dato-DXd combination with anti-mSIRPα Ab induced PD-1 expression on CD8^+^ T cells (Fig 5C), providing a rationale for triplet combinations of Dato-DXd, anti-SIRPα Ab, and anti-PD-1/PD-L1 Ab, which would be explored in future preclinical studies.

The data together with others shed light on the molecular mechanism underpinning the anti-tumor activity of anti-CD47 and anti-SIRPα Abs. In the cancer vaccination model, anti-mSIRPα Ab following DXd-treated CT26.WT vaccination resulted in the activation of anti-tumor T cells, while anti-mCD47 Ab did not (Fig 4B). This was consistent with the recent reports showing anti-tumor efficacy of this anti-mCD47 Ab (clone MIAP410) but not anti-SIRPα Ab is independent of T cells. Combination of radiotherapy with anti-mCD47 Ab in immunocompetent mice bearing subcutaneous tumor showed enhanced anti-tumor efficacy, which remained the same when CD8^+^ T cells were depleted [45]. On the other hand, combination of radiotherapy with anti-mSIRPα Ab showed enhanced anti-tumor efficacy, which was dependent on CD8^+^ T cells [46]. Further studies are needed to address the difference between anti-CD47 and anti-SIRPα Abs in the T cell dependency. Apart from its role in suppressing macrophages, CD47 has been found to have various other functions [9]. For instance, CD47 has shown to be essential for the survival of T cells in mouse and human chimeric-antigen receptor T cells [47, 48]. In mice, CD47 deficiency hindered the anti-viral response of natural killer cells [49]. Further fundamental research as well as clinical trials exploring anti-CD47 and anti-SIRPα Ab would help differentiate the role of CD47 and SIRPα in human biology.

The results suggest anti-SIRPα Ab may show combination effects with other DXd-ADCs beyond Dato-DXd and T-DXd. Dato-DXd and T-DXd induced ICD, which occurred via the DXd payload, not the Ab portion (Fig 3 and S7 Fig in S1 File). In the vaccination model using DXd payload, activation of tumor-specific T cells was induced by combination of DXd-treated CT26.WT vaccination with anti-mSIRPα Ab treatment, but not in the absence of anti-mSIRPα Ab (Fig 4B), suggesting the addition of anti-mSIRPα Ab was requisite for the T cell activation, presumably via augmentation of phagocytosis against the vaccine component. Future studies will determine whether anti-SIRPα Ab shows benefit to other DXd-ADCs. Since ICD can be induced by ADC payloads other than DXd, it is of interest to speculate that anti-SIRPα Ab may synergize with other ADCs with ICD-enabling payloads. In this regard, T-DM1 and other ADC payloads have shown to induce ICD [33, 50], setting a stage for anti-SIRPα Ab to combine with a spectrum of ADCs as a novel combination therapy covering multiple solid tumors.

There are limitations associated with this study. Firstly, the exogenously expressed human HER2 and TROP2 on mouse cancer cells might make the tumor vulnerable to existing immunity. While this was necessary to assess the synergy between DXd-ADCs and anti-mSIRPα Ab in immunocompetent animals, the model could potentially represent a "more treatable" cancer scenario. Secondly, the animal studies employed a subcutaneous tumor implant model. Although this model is commonly used in immuno-oncology drug testing, it may not fully capture the complexity and heterogeneity of human solid tumors. For this regard, further studies utilizing additional preclinical models, such as orthotopic tumor implant models and genetically engineered mouse models, are needed to validate the findings in a broader context. Thirdly, we employed an anti-mSIRPα Ab as a surrogate for DS-1103a, since DS-1103a did not cross-react to mSIRPα. While surrogate antibodies are commonly used in non-clinical studies to investigate mechanisms of action and concepts in mouse models, it is important to note that surrogate antibodies may possess different characteristics when compared to their corresponding parental antibodies intended for human use. To bridge this gap, ADCP assays were conducted using DS-1103a and human cancer cells. Comprehensive insights into this matter could be gained through additional investigations, such as clinical trials and potentially tumor tissue assays.

As a summary, this study has shown novel combination therapies of two DXd-ADCs with anti-SIRPα Ab. Anti-SIRPα Ab successfully enhanced the ADCP of both Dato-DXd and T-DXd *in vitro* and synergized with DXd-treated whole cell cancer vaccine. Furthermore, combination of anti-mSIRPα Ab with Dato-DXd or T-DXd showed enhanced anti-tumor activity in syngeneic models. These data provide a fundamental basis for the combination treatment in various cancer patients. DS1103-074 trial is now ongoing to test the hypothesis in the clinical setting (NCT05765851).

## Supporting information

S1 File (DOCX)
