# Supplementary material for: Blockade of SIRPα-CD47 axis by anti-SIRPα antibody enhances anti-tumor activity of DXd antibody-drug conjugates
Source: PLoS One. 2024 Jun 6;19(6):e0304985. doi: 10.1371/journal.pone.0304985 (PMC11156334; doi:10.1371/journal.pone.0304985)
Supplement: S1 File — (DOCX) [file pone.0304985.s001.docx]

**Supporting information**

**Blockade of SIRPα-CD47 axis by anti-SIRPα antibody enhances anti-tumor activity of DXd-ADCs**

Mayumi Sue^1^, Takuya Tsubaki^2^, Yoko Ishimoto^3^, Shinko Hayashi^1^, Saori Ishida^1^, Takafumi Otsuka^4^, Yoshitaka Isumi^1^, Yumi Kawase^5^, Junko Yamaguchi^6^, Takashi Nakada^7^, Jun Ishiguro^5^, Kensuke Nakamura^8^, Reimi Kawaida^5^, Toshiaki Ohtsuka^5^, Teiji Wada^1^, Toshinori Agatsuma^9^, Norihito Kawasaki^1*^

^1^Discovery Research Laboratories II, ^2^Modality Research Laboratories III, ^3^Translational Science Department I, ^4^Research Innovation Planning Department, ^5^Discovery Research Laboratories V, ^6^Discovery Research Laboratories I, ^7^Modality Research Laboratories I, ^8^Modality Research Laboratories II, ^9^R&D Division, Daiichi Sankyo Co., Ltd., Tokyo, Japan

*Corresponding author: [kawasaki.norihito.yt@daiichisankyo.co.jp](mailto:kawasaki.norihito.yt@daiichisankyo.co.jp) (NK)

| **S1 Table. Cell lines used in this study.** | | | |
| --- | --- | --- | --- |
| Name | Provider | Catalog number | Culture medium used |
| CHO-K1 | DS Pharma Biomedical, Osaka, Japan | EC85051005-F0 | Ham’s F-12K medium (Thermo Fisher Scientific, Waltham, MA) supplemented with 10% heat-inactivated fetal bovine serum (FBS, GE Healthcare, Chicago, IL) |
| LK-2 | Health Science Research Resource, Osaka, Japan | JCRB0829 [1] | RPMI 1640 (Thermo Fisher Scientific) supplemented with 10% heat-inactivated FBS (R10) |
| HCC827 | American Type Culture Collection (ATCC), Manassas, VA | CRL-2868 | R10 |
| CT26.WT | ATCC | CRL-2638 | R10 |
| MC38 | National Cancer Institute | N/A | R10 |
| SK-BR-3 | ATCC | HTB-30 | McCoy's 5A Medium (Thermo Fisher Scientific) supplemented with 10% heat-inactivated FBS |
| hHER2 CT26.WT | Daiichi Sankyo, Tokyo, Japan | N/A, generated as previously described [2] | R10 supplemented with 250 μg/mL Geneticin (Thermo Fisher Scientific) |
| hTROP2 MC38 | Daiichi Sankyo, Tokyo, Japan | N/A, generated using pQCXIN vector encoding human TROP2 (NM_002353.3) | R10 |

[1] Yoshioka S. Studies on thiol protease inhibitor isolated from human lung cancer cell line. Hiroshima J Med Sci 1989; 37: 199-215.

[2] Iwata TN, Ishii C, Ishida S, Ogitani Y, Wada T, Agatsuma T. A HER2-Targeting Antibody-Drug Conjugate, Trastuzumab Deruxtecan (DS-8201a), Enhances Antitumor Immunity in a Mouse Model. Mol Cancer Ther 2018;17(7):1494-503 <https://doi.org/10.1158/1535-7163.MCT-17-0749> PMID: 29703841

| **S2 Table. Commercially available antibodies used in this study.** | | | |
| --- | --- | --- | --- |
| Antibody | Provider | Clone | Dilution used |
| Ultra-LEAF purified mouse IgG1 (isotype control for anti-mSIRPα Ab) | BioLegend | MOPC-21 | Used in S1 Fig as indicated |
| Ultra-LEAF purified human IgG1 (isotype control for trastuzumab and datopotamab) | BioLegend | QA16A12 | Used in Fig 2 and S5 Fig as indicated |
| Trastuzumab | CHUGAI PHARMACEUTICAL | N/A | Used in Fig 6 and S5 and S6 Figs as indicated |
| Peroxidase-AffiniPure F(ab’)2 Fragment Goat Anti-Human IgG, Fc gamma Fragment Specific | Jackson ImmunoResearch | N/A (Cat # 109-036-098) | 1:5000 |
| Peroxidase AffiniPure Goat Anti-Mouse IgG, Fcγ subclass 1 specific | Jackson ImmunoResearch | N/A (Cat # 115-035-205) | 1:10000 |
| Anti-Human IgG (Fc) antibody | Cytiva | N/A (Cat # BR100839) | 1:20 |
| Anti-mCD47 | Bio X Cell | MIAP410 | Used in Fig 4 as indicated |
| BD Fc Block human | BD Biosciences | N/A (Cat # 564220) | 1:50 |
| TruStain fcX (anti-mouse CD16/32) | BioLegend | 93 | 1:100 |
| Anti-mouse Ly-6G/Ly-6C (Gr-1)-Brilliant violet (BV) 421 | BioLegend | RB6-8C5 | 1:100 |
| Anti-mouse CD11c Ab-PerCP-Cy5.5 | BioLegend | N418 | 1:40 |
| R-Phycoerythrin (PE) goat anti-mouse IgG | BioLegend | N/A (Cat # 405307) | 1:100 |
| Anti-mCD8α-Brilliant ultraviolet (BUV)395 | BD Biosciences | 53-6.7 | 1:100 |
| Anti-mCD45-Pacific Blue (PB) | BioLegend | 30-F11 | 1:200 |
| Anti-mCD3-FITC | BioLegend | 17A2 | 1:100 |
| Anti-mCD4-PE | BD Biosciences | RM4-5 | 1:100 |
| Anti-human/mouse Granzyme B (GzmB)-PE-Cy7 | BioLegend | QA16A02 | 1:100 |
| Mouse IgG1 isotype control Ab-PE-Cy7 | BD Biosciences | MOPC-21 | 1:50 |

| **S2 Table. continued** | | | |
| --- | --- | --- | --- |
| Anti-mPD-1-Alexa Fluor 647 (AF647) | BioLegend | RMP1-30 | 1:50 |


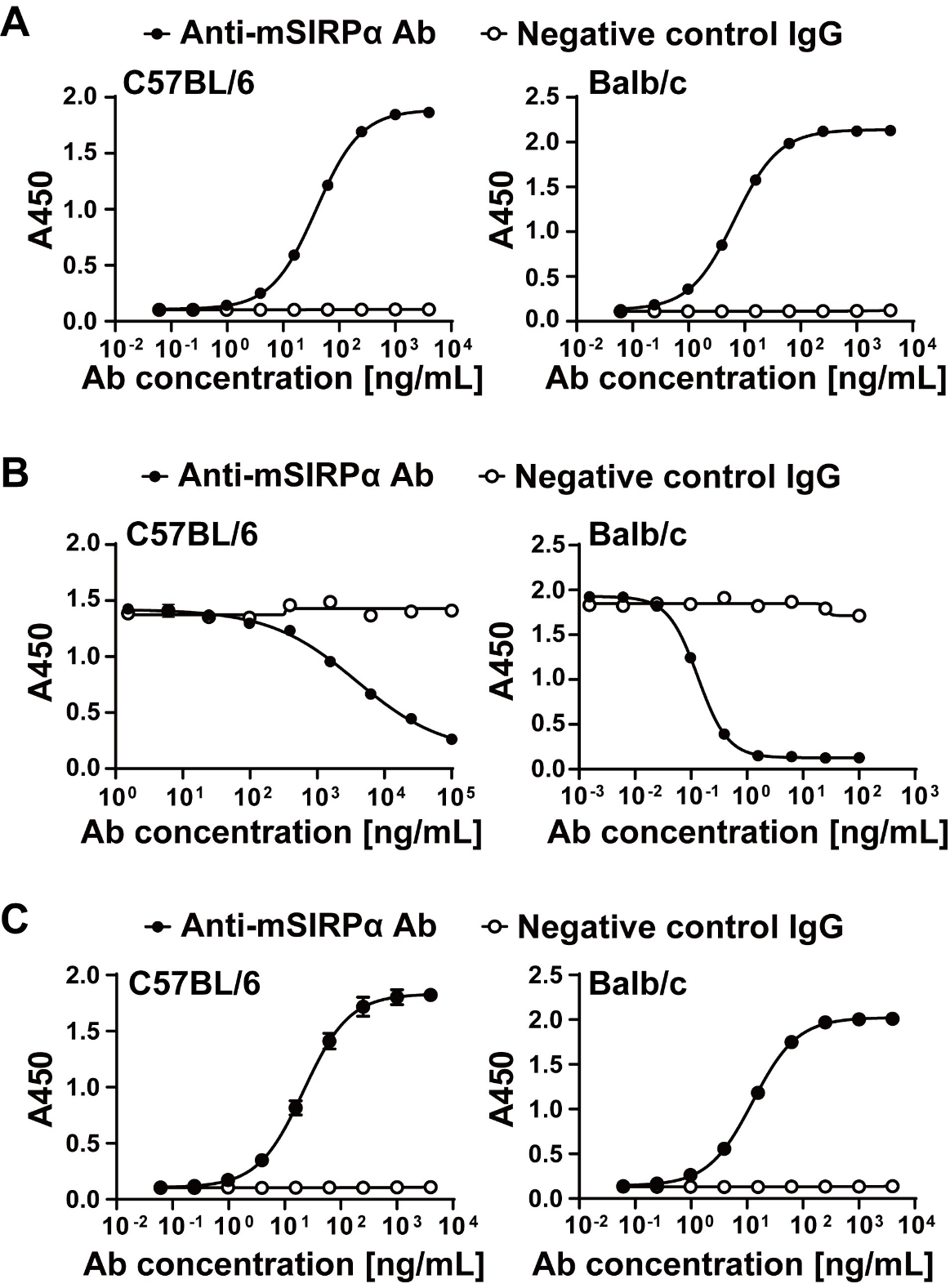


**S1 Fig.** **Anti-mSIRPα Ab binds to mSIRP family proteins and inhibits mSIRPα-mCD47 interaction.** (**A**) The binding of anti-mSIRPα Ab to recombinant mouse SIRPα of C57BL/6 and Balb/c strains was measured as absorbance at 450 nm (A450) by ELISA assay. (**B**) Inhibition of mCD47-Fc binding to recombinant mouse SIRPα by anti-mSIRPα Ab was measured in a competitive ELISA assay. (**C**) The binding of anti-mSIRPα Ab to recombinant mouse SIRPβ1 of C57BL/6 and Balb/c strains was measured as in (A). Data represents the mean ± standard deviation (three technical replicates).


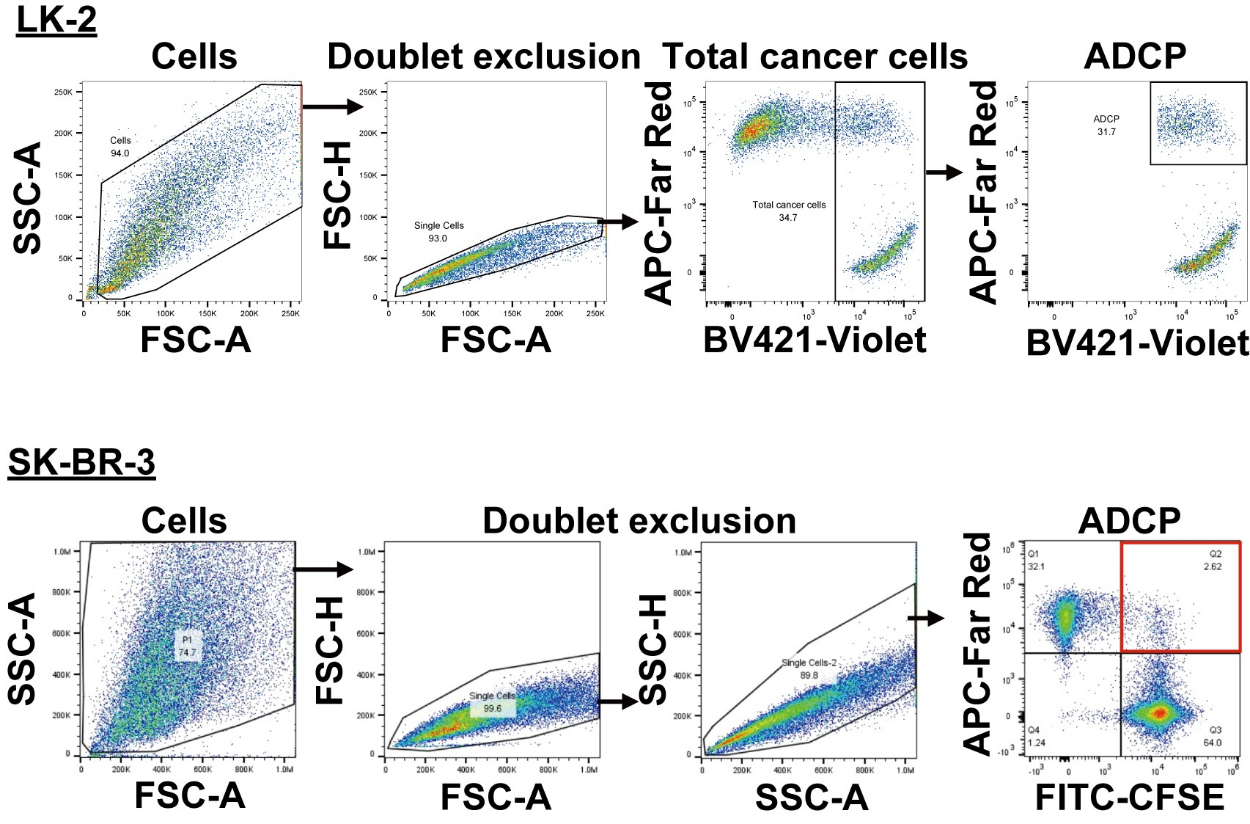


**S2 Fig.** **ADCP gating used in this study.** Gating strategy used to identify phagocytosed cancer cells is shown for Fig 2B (LK-2 cells) and 6A (SK-BR-3 cells), respectively.


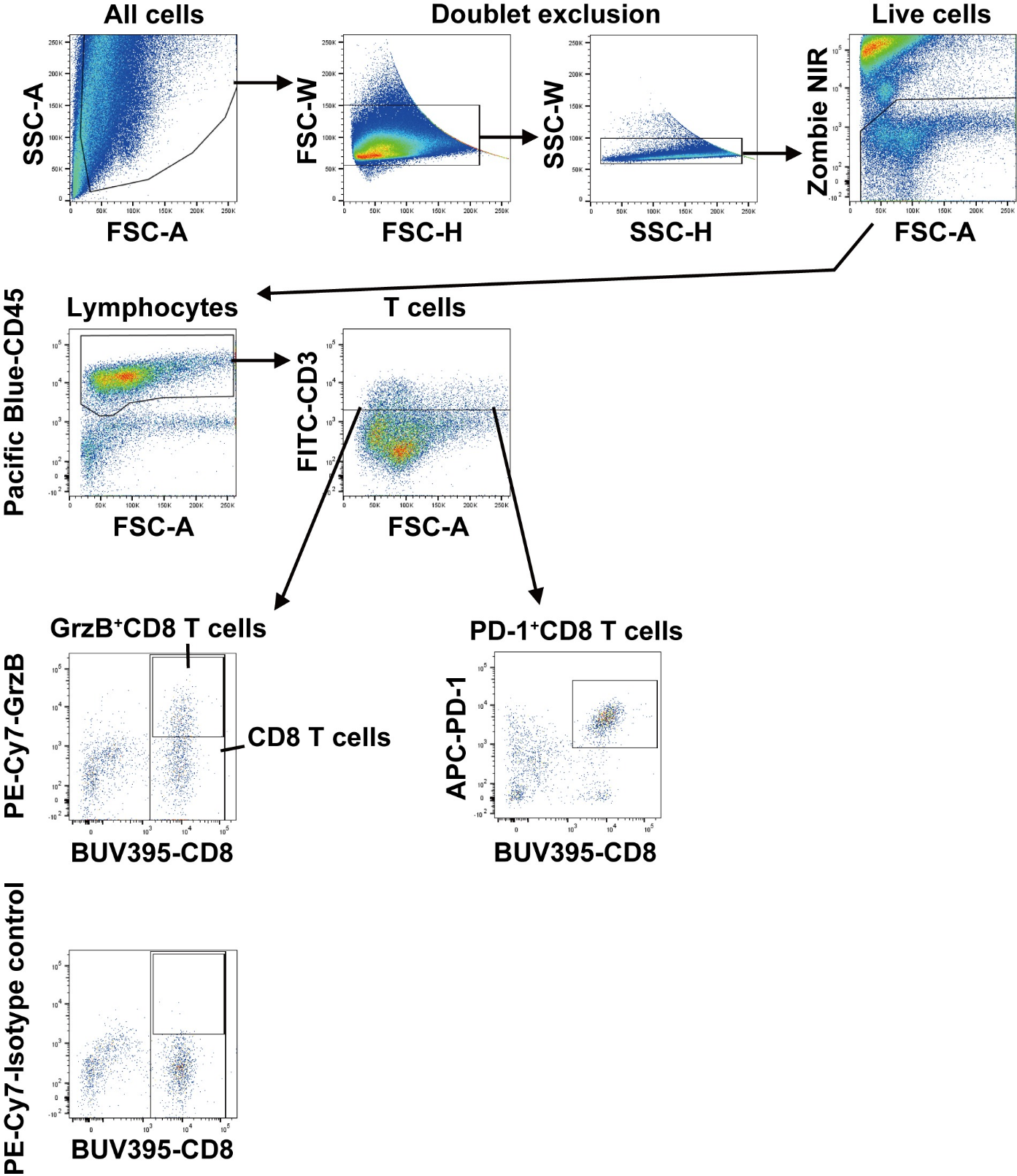


**S3 Fig.** **Gating strategy for TIL analysis.** Gating strategy to identify CD8^+^ T, GrzB^+^ CD8^+^ T, and PD-1^+^ CD8^+^ T cells in Fig 5B is shown.


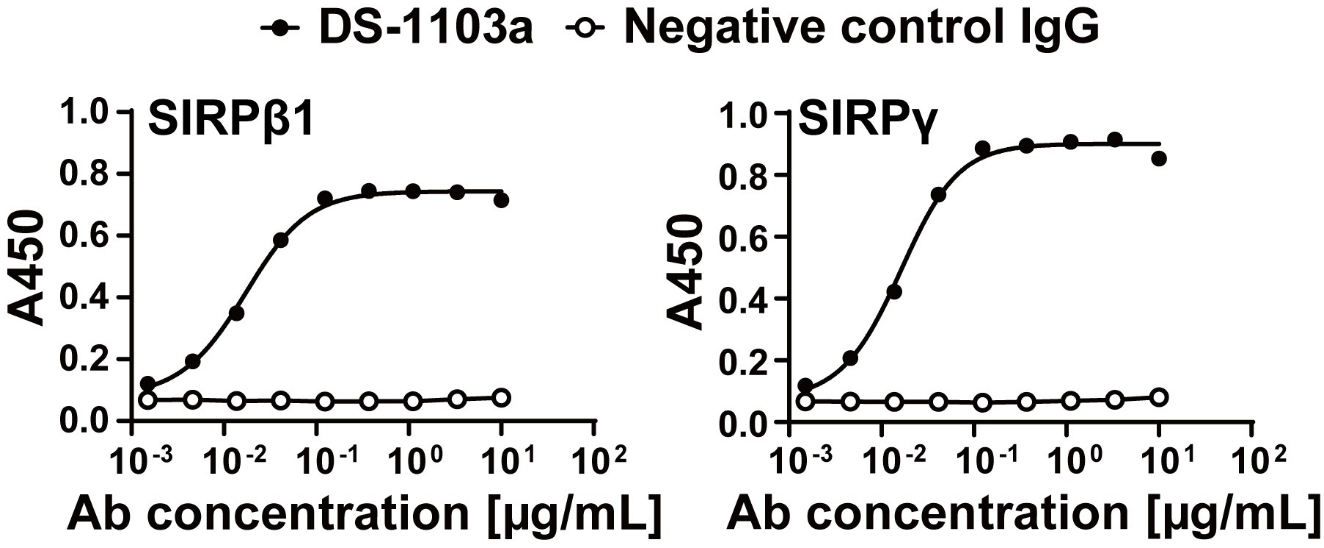


**S4 Fig.** **DS-1103a binds to human SIRPβ1 and γ.** The binding of DS-1103a to human SIRPβ1 and γ was measured as absorbance at A450 by cell-based binding assay using CHO-K1 cells transiently transfected with an expression plasmid for each human SIRP family protein. Data represent the mean ± standard error (three technical replicates).


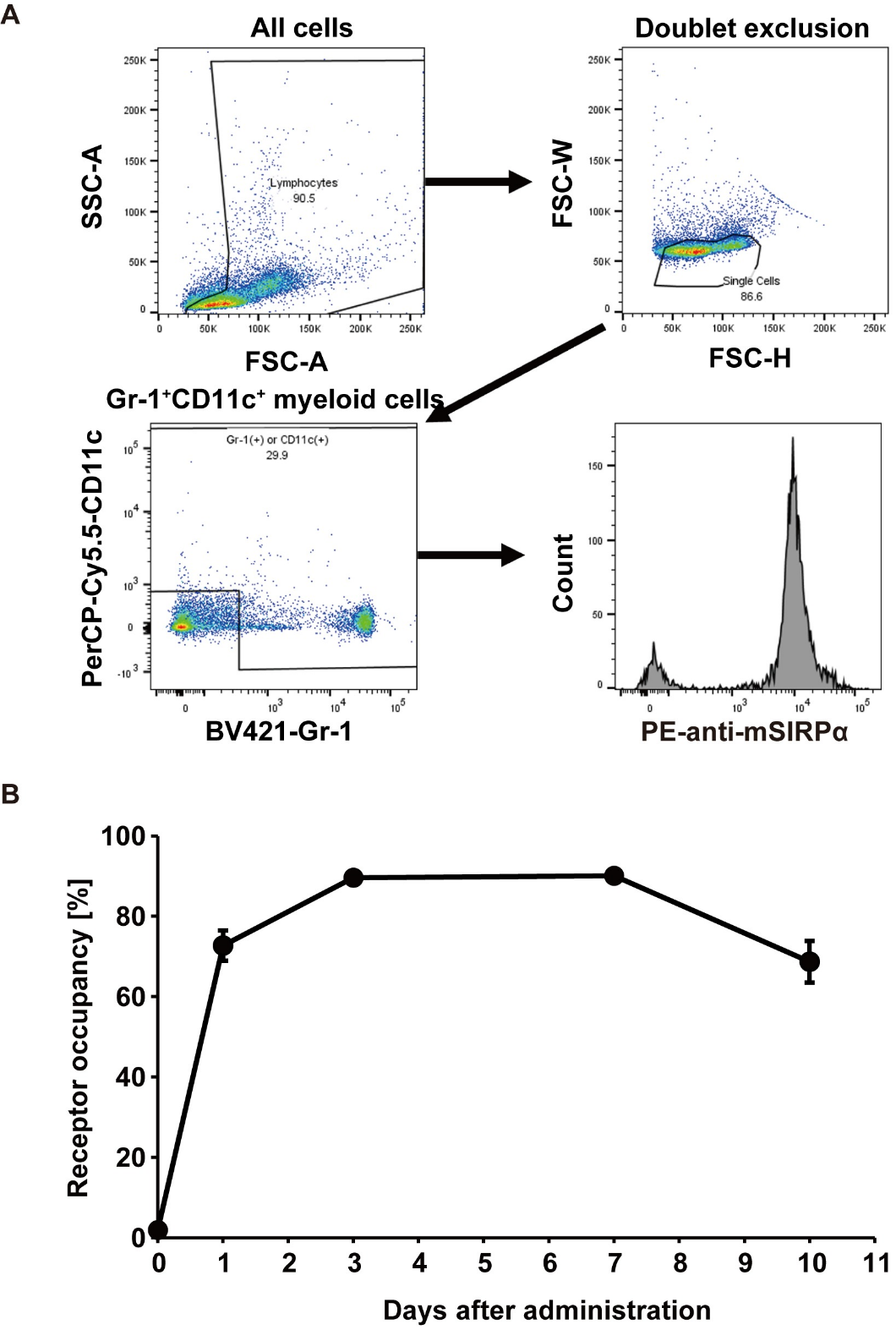


**S5 Fig.** **Time-course analysis of SIRPα receptor occupancy of anti-mSIRPα Ab on peripheral blood myeloid cells after single administration to mice.** The anti-mouse SIRPα surrogate Ab (10 mg/kg) was intraperitoneally administered to BALB/c mice on day 0. (**A**) Just before administration, and on 1, 3, 7, and 10 days after administration, peripheral blood samples were collected and stained with the indicated Abs and analyzed by flow cytometry. (**B**) SIRPα-receptor occupancy of anti-mouse SIRPα Ab on peripheral blood myeloid cells from each mouse is shown as the mean ± SEM (n = 3 at each time point).

**
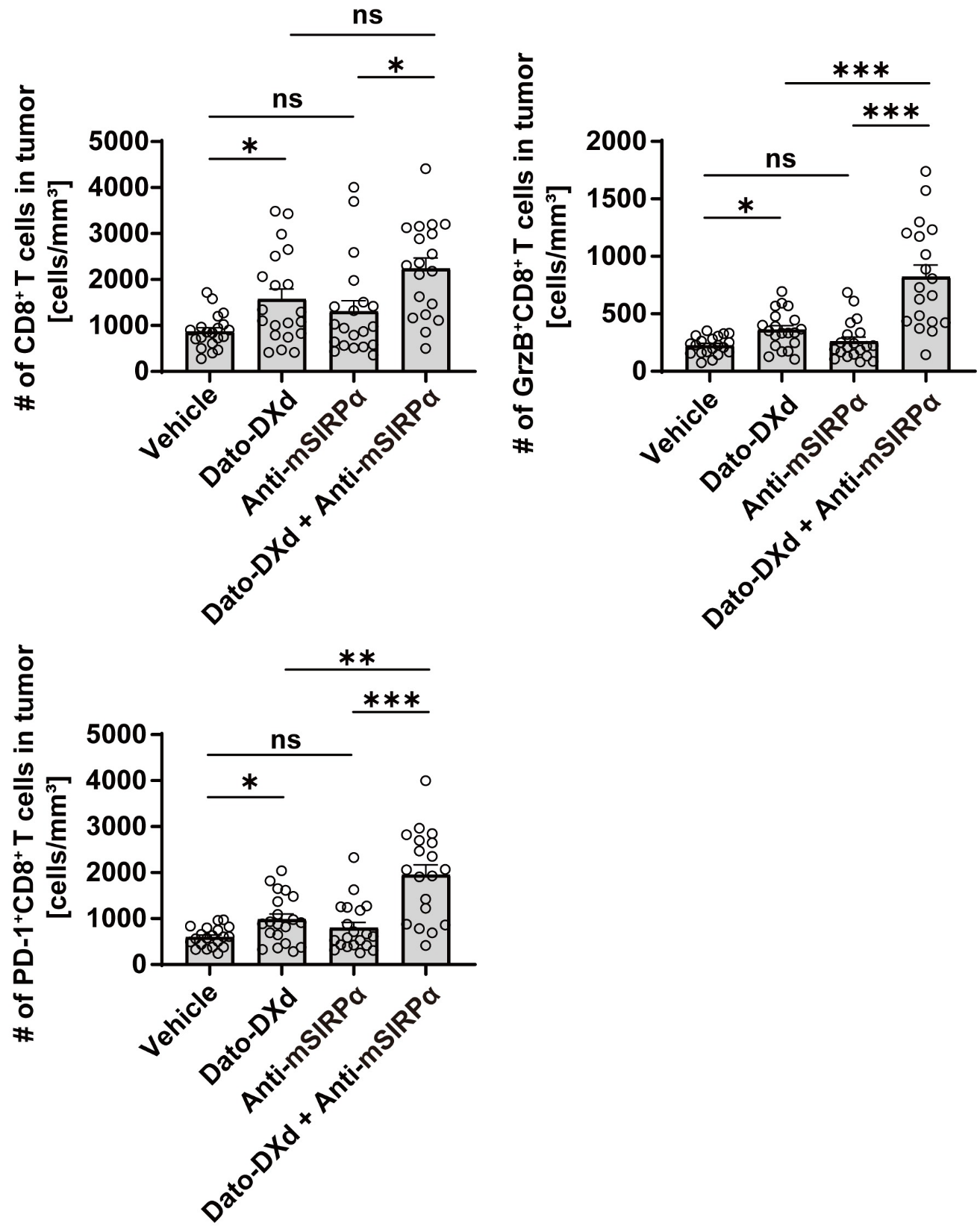
**

**S6 Fig.** **Number of T cells in the tumor in TIL analysis in Fig 5.** The numbers of CD8^+^ T cells, GrzB^+^ CD8^+^ T cells, PD-1^+^ CD8^+^ T cells per tumor volume [cells/mm^3^] from each tumor sample are shown with the mean ± SEM of each group (combination group, n = 19; other groups, n = 20). P values for the numbers were determined by parametric Dunnett’s multiple comparison test.

**
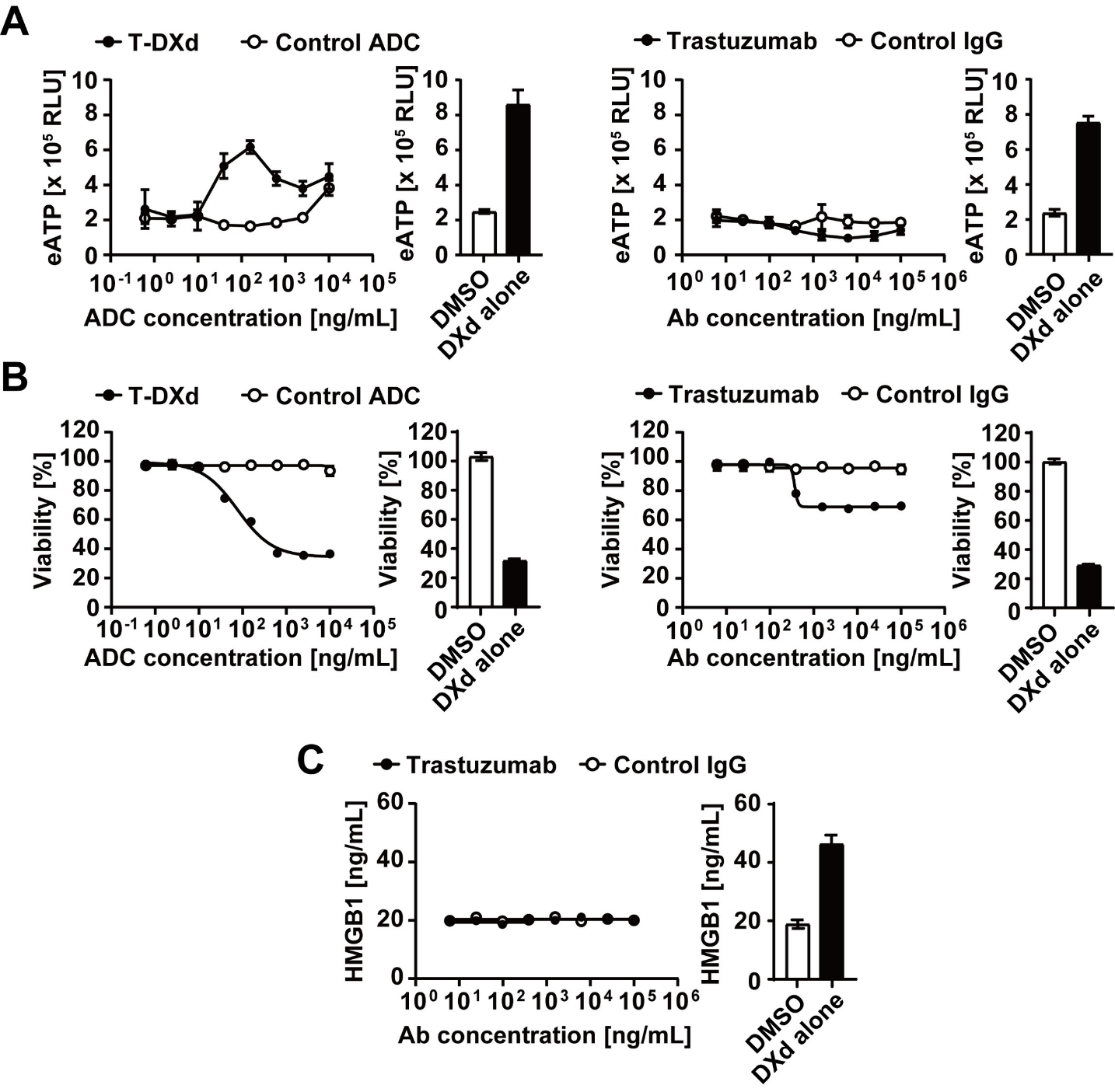
**

**S7 Fig.** **T-DXd but not trastuzumab induces ICD of SK-BR-3 cells.** SK-BR-3 cells were cultured with the various concentrations of T-DXd, trastuzumab, or the negative controls for 4 days. (**A**) eATP secretion was measured by a luminescence-based assay. Viability of SK-BR-3 cells (**B**) and HMGB1 secretion (**C**) were measured as in Fig. 3. The data represent the mean ± SD (three technical replicates).


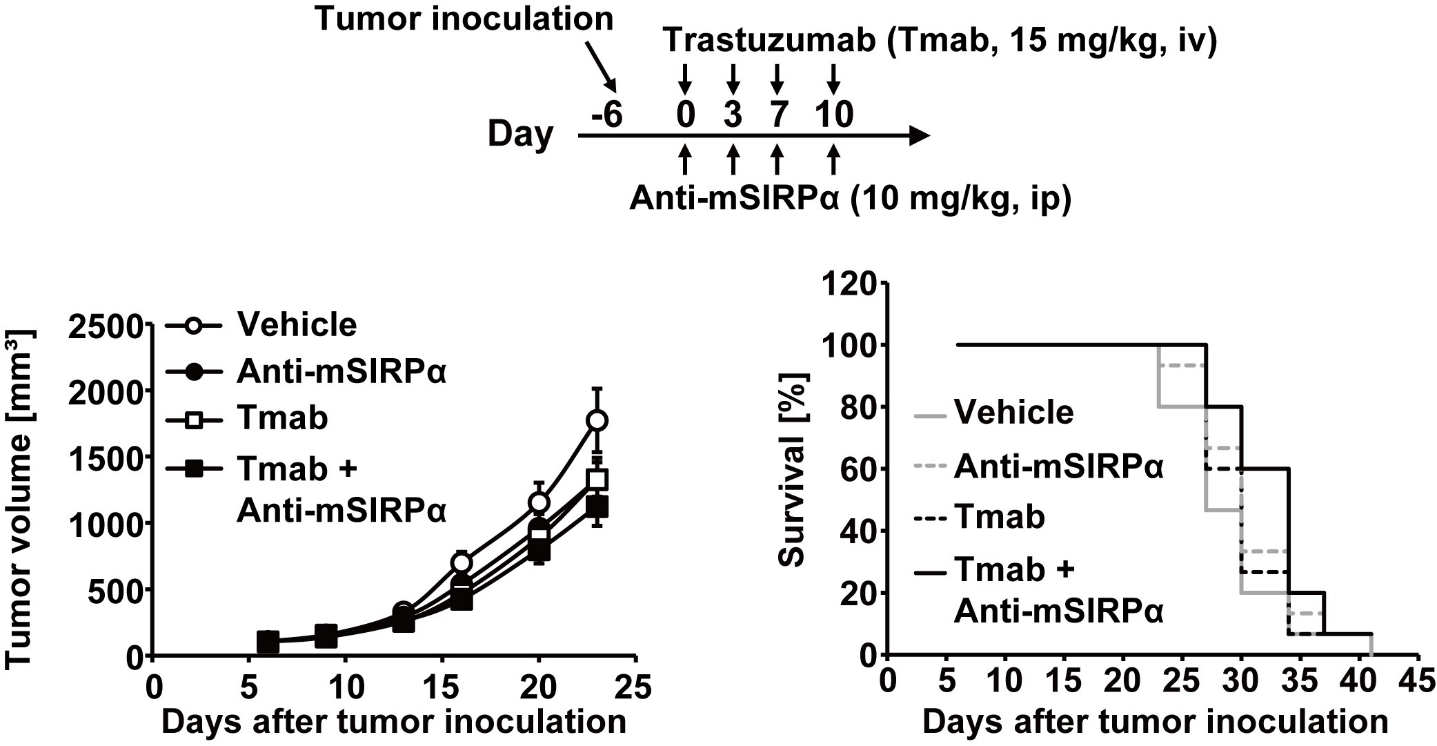


**S8 Fig.** **Combination of Trastuzumab with anti-mSIRPα Ab failed to show enhanced anti-tumor activity.** hHER2-CT26.WT cells (3 × 10^6^ cells/head) were subcutaneously inoculated into the right flank of BALB/c mice on day 0. The indicated reagents were administered as a monotherapy or in the indicated combinations, starting on day 6. Tumor volumes are shown as mean ± SEM (n = 15). Representative data from two independent experiments are shown.
